# Synergism Among the Four Tobacco Bushy Top Disease Casual Agents in Symptom Induction and Aphid Transmission

**DOI:** 10.3389/fmicb.2022.846857

**Published:** 2022-04-04

**Authors:** Xiaojiao Chen, Hengming Luo, Jingyi Zhang, Yan Ma, Kehua Li, Feng Xiong, Yahui Yang, Jiazhen Yang, Pingxiu Lan, Taiyun Wei, Yi Xu, Hairu Chen, Fan Li

**Affiliations:** ^1^State Key Laboratory for Conservation and Utilization of Bio-Resources in Yunnan, Yunnan Agricultural University, Kunming, China; ^2^Key Laboratory of Agricultural Biotechnology of Yunnan Province, Institute of Biotechnology and Germplasm Resources, Yunnan Academy of Agricultural Sciences, Kunming, China; ^3^Institute of Plant Virology, Fujian Agriculture and Forestry University, Fuzhou, China; ^4^Department of Plant Pathology, Nanjing Agricultural University, Nanjing, China

**Keywords:** tobacco bushy top virus, tobacco vein distorting virus, TBTVsatRNA, TVDV-associated RNA, synergism, aphid transmission

## Abstract

Tobacco bushy top disease (TBTD), caused by multiple pathogens including tobacco bushy top virus (TBTV), tobacco vein distorting virus (TVDV), TBTV satellite RNA (TBTVsatRNA), and TVDV-associated RNA (TVDVaRNA), is a destructive disease in tobacco fields. To date, how these causal agents are co-transmitted by aphid vectors in field and their roles in disease symptom induction remain largely unknown, due mainly to the lack of purified causal agents. In this study, we have constructed four full-length infectious clones, representing the Yunnan Kunming isolates of TVDV, TBTV, TBTVsatRNA, and TVDVaRNA (TVDV-YK, TBTV-YK, TBTVsatRNA-YK, and TVDVaRNA-YK), respectively. Co-inoculation of these four causal agents to tobacco K326 plants caused typical TBTD symptoms, including smaller leaves, necrosis, and plant stunting. In addition, inoculation of tobacco K326 plants with TBTV alone caused necrosis in systemic leaves by 7 dpi. Tobacco K326 and *Nicotiana benthamiana* plants infected by single virus or multiple viruses showed very different disease symptoms at various dpi. RT-PCR results indicated that co-infection of TVDVaRNA-YK could increase TVDV-YK or TBTV-YK accumulation in *N. benthamiana* plants, suggesting that TVDVaRNA-YK can facilitate TVDV-YK and TBTV-YK replication and/or movement in the infected plants. Aphid transmission assays showed that the successful transmission of TBTV-YK, TBTVsatRNA-YK, and TVDVaRNA-YK by *Myzus persicae* depended on the presence of TVDV-YK, while the presence of TBTVsatRNA-YK increased the aphid transmission efficiency of TBTV and TVDV. We consider that these four new infectious clones will allow us to further dissect the roles of these four causal agents in TBTD induction as well as aphid transmission.

## Introduction

Tobacco bushy top disease (TBTD) is one of the most damaging diseases in tobacco fields in the Yunnan Province of China, and also in other Africa countries, and is characterized by the bushy and stunting phenotypes. Many TBTD-infected tobacco plants also show smaller leaves, leaf distortion, leaf curling, and/or yellowing. TBTD was first reported in Zimbabwe in 1958, and then in Malawi, Zimbabwe, Pakistan, and Thailand, based on the symptoms and vector transmission mode (Gates, 1962; Blancard et al., 1999). In 1993, an outbreak of TBTD was observed in the tobacco fields in the western region of the Yunnan Province. Since then, TBTD has become the major threat to tobacco productions in the Yunnan Province (Gu and Chen, 1994; Li et al., 2005). The causal agents of TBTD in China are known to be tobacco bushy top virus (TBTV), a member in the genus *Umbravirus* (Mo et al., 2003); tobacco vein distorting virus (TVDV), a member in the genus *Polerovirus* (Mo et al., 2010); tobacco bushy top virus satellite RNA (TBTVsatRNA); and tobacco vein distorting virus-associated RNA (TVDVaRNA; Mo et al., 1998, 2011). In a recent study, TBTD causal agents were also found to infect 10 cash crops, including tomato, broad bean, pea, pumpkin, and oilseed rape (Tan et al., 2021). In Liu et al. (2014) reported that the infected tobacco plants showing typical TBTD symptoms often contained more than one causal agents. Although the symptoms of the TBTD plants reported in China were similar to that of the TBTD plants reported in Ethiopia, the causal agents of TBTD plants in Ethiopia were Ethiopian tobacco bushy top virus (ETBTV, an umbraviruses), potato leafroll virus (PLRV, a polerovirus), and a ETBTV satellite RNA (ETBTVsatRNA; Abraham et al., 2014). In addition to tobacco, mixed virus infection has also been found in other plant species. For example, carrot plants were reported to be co-infected with carrot red leaf virus (CtRLV, a polerovirus; Huang et al., 2005), carrot mottle virus (CMoV, an umbravirus; Watson et al., 1964; Murant et al., 1969) and/or carrot mottle mimic virus (CMoMV, an umbravirus) with CtRLV associated RNA (CtRLVaRNA; Gibbs et al., 1996; Yoshida, 2020). Groundnut rosette disease (GRD) was also reported to be caused by groundnut rosette umbravirus (GRV), GRV satellite RNA (GRVsat-RNA), and groundnut rosette assistor virus (GRAV, a polerovirus; Reddy et al., 1985). To date, the synergistic effect caused by umbravirus and polerovirus co-infection in plants remains largely unknown (Reddy et al., 1985; Naidu et al., 1998; Yoo et al., 2017; Yoshida, 2020). In 2021, our laboratory reported that TBTV and several other viruses could co-infect tomato and pepper plants, resulting in complete failures of crop productions in some fields (Li et al., 2021).

Tobacco bushy top virus genome is a positive-sense single-stranded RNA with 4,152 nucleotides (nt) and has four open reading frames (ORFs). TBTV ORF1 encodes a 35 kDa P1 protein, and the ORF2 is overlapped with the 3′ end sequence of ORF1 and encodes a putative 98 kDa fusion through a −1 ribosomal frameshift. The ORF3 encodes a putative 26 kDa protein, and the ORF4 encodes a 27 kDa protein. Like other umbraviruses, TBTV is sap transmissible. In the presence of a helper luteovirus (e.g., TVDV), TBTV can also be transmitted by *Myzus persicae* (green peach aphid) to tobacco plants in a circulative, non-propagative manner (Chen and Feng, 2006; Mo et al., 2010).

Tobacco vein distorting virus is also a positive-sense single-stranded RNA virus with 5,920 nts and is encapsidated in 25–30 nm icosahedral particles. The genome RNA of TVDV contains seven ORFs and encode seven proteins. The ORF0 and ORF1 encodes a 28 kDa P0 protein and a 72 kDa P1 protein, respectively. The ORF2 is overlapped with the ORF1 and is predicted to be translated by fusion with the product of ORF1 through ribosomal frameshifting. The ORF3a, ORF3, ORF4, and ORF5 are located in the 3′ half of the genomic RNA and produce a subgenomic RNA. Translation of ORF3a is predicted to be dependent on a non-AUG initiation, and the ORF3 encodes a 22 kDa CP protein. The ORF4, completely embedded in ORF3 but in a different reading frame, encodes a 17 kDa MP protein, and the ORF5 encodes a 80 kDa readthrough protein, *via* readthrough of the stop codon of ORF3 (Mo et al., 2010; Xu et al., 2018). Several reports have indicated that TVDV and TBTV co-infection in tobacco plants could cause bushy top-like disease symptoms, while TVDV and tobacco mottle virus co-infection in tobacco plants causes rosette-like disease symptoms (Smith, 1946; Mo et al., 2002). Previous studies have indicated that TVDV cannot be transmitted to its host plants through mechanical inoculation, even in the presence of TBTV (Gu and Chen, 1994; Li et al., 2005; Mo et al., 2010).

TBTVsatRNA and TVDVaRNA are single-stranded RNAs with 824 and 2,971 nts (with two ORFs), respectively. The ORF1b of TVDVaRNA encodes an RNA-dependent RNA polymerase, probably *via* the readthrough of the stop codon of ORF1a (Mo et al., 2011). TBTVsatRNA and TVDVaRNA were identified as the causal agents of TBTD by Mo et al. (1998, 2011). To date, many satellite RNAs have been found in virus-infected plants. Replications of virus satellite RNAs are known to rely on their helper viruses (Simon et al., 2004). On the other hand, the presence of GRV satellite RNA was necessary for the induction of rosette-like disease symptoms, GRV replication, and aphid transmission of GRV (Taliansky and Robinson, 1997). Cucumber mosaic virus (CMV) satRNA was also found to affect the CMV-induced symptoms in specific host plants (Shimura et al., 2011; He et al., 2019). The function of TVDVaRNA in infected plants was speculated to be similar to that reported for other tombusvirus-like associated RNAs (tlaRNAs), based on their genome structure similarities.

Many plant virus infectious clones have been constructed in recent years and used to investigate the arms races between viruses and their host plants (Wang et al., 2015; Gao et al., 2019; Feng et al., 2020). In addition, many plant virus infectious clones have been further modified and used to investigate gene functions in dicotyledonous and/or monocotyledonous plants through reverse genetics (Liu et al., 2002; Ding et al., 2006, 2018; Sha et al., 2014; Abrahamian et al., 2020). Although the full-length sequences of TBTV, TVDV, TVDVaRNA, and TBTVsatRNA have been published previously, no infectious clones representing these four causal agents have been constructed. In this paper, we report the complete nucleotide sequences of the Yunnan Kunming isolates of TBTV, TVDV, TBTVsatRNA, and TVDVaRNA collected from the tobacco fields (referred to as TBTV-YK, TVDV-YK, TBTVsatRNA-YK, and TVDVaRNA-YK), and constructed infectious clones, representing each of the four causal agents. The infectivities of these four clones were confirmed through Agrobacterium-mediated infiltration into tobacco and *Nicotiana benthamiana* plants. Our results show that TVDVaRNA-YK can increase the accumulation levels of TBTV-YK and TVDV-YK. Aphid transmission efficiencies of TVDV-YK, TBTV-YK, TBTVsatRNA-YK, and TVDVaRNA-YK were also tested, individually or in various combinations, using *M. persicae* as the vector.

## Materials and Methods

### Virus Source

Infected tobacco plants showing typical TBTD symptoms were collected from tobacco fields in the Kunming City, Yunnan Province, and maintained inside an insect-proof greenhouse. Causal agents of these infected plants were analyzed using the next-generation RNA sequencing (NGS) technology at the Biomarker Technologies (Beijing, China). The sequencing data were processed using the method described previously (Tan et al., 2021), and the full-length causal agent sequences were named as TVDV Yunnan Kunming isolate (TVDV-YK, GenBank accession number OMO62616), TBTV Yunnan Kunming isolate (TBTV-YK, OMO62615), TBTV Yunnan Kunming satellite RNA (TBTVsatRNA-YK, OMO62618), and TVDV Yunnan Kunming associated RNA (TVDVaRNA-YK, OMO62617), respectively. To facilitate writing, the isolate YK2 mentioned below will be replaced by YK and the four causal agent sequences are list in Supplementary Table 1.

### Phylogenetic Analysis

The resulting causal agent sequences were used to blast search and align with the relevant sequences in the GenBank database using the MUltiple Sequence Comparison by Log-Expectation (MUSCLE) software. The evolutionary relationships among the related viruses were determined using the neighbor-joining (NJ) method in the MEGA7 software with 1,000 bootstrap replicates.

### Constructions of Infectious Clones

To investigate the roles of individual causal agents on TBTD induction in plants, we constructed four full-length infectious clones, representing TBTV-YK, TBTVsatRNA-YK, TVDV-YK, and TVDVaRNA-YK, respectively. Primers used to amplify the four causal agent sequences are listed in Supplementary Table 2. The reverse transcriptions (RT) were performed using the PrimeScript II 1^st^ strand cDNA Synthesis Kit (TaKaRa Biotechnology, Dalian, China), and the polymerase chain reactions (PCR) were performed using the PrimeSTAR® Max DNA Polymerase as previously described (Tan et al., 2021). The amplified PCR products were gel purified and inserted between the *Sma*I and *Stu*I cloning site in the binary vector pB301-2X35S-MCS-HDVRZ-NOS-1 using the In-Fusion® HD Cloning Kit as instructed (TaKaRa Biotechnology, Dalian, China). The resulting plasmids were individually propagated in *Escherichia coli* DH5α competent cells, sequenced, and then transformed into *Agrobacterium tumefaciens* strain EHA105. The transformed Agrobacterium cells were selected on the Luria–Bertani (LB) medium plates supplemented with Kanamycin and Rifampicin (50 mg/L each) for 48 h at 28°C followed by further propagation prior to plant inoculation.

### Plant Inoculation

*Nicotiana benthamiana* and tobacco cv. K326 plants (referred to tobacco K326 pants thereafter) were grown inside a greenhouse maintained at 25/23°C (day/night) and a 16/8 h (day/night) photoperiod. To determine the infectivities of these four infectious clones, the overnight grown Agrobacterium cultures were infiltrated, individually or in various combinations, into fully expanded leaves of *N. benthamiana* or tobacco K326 plants. The plants agro-infiltrated with an Agrobacterium culture containing pCB301 (an empty cloning vector) were used as controls. The infiltrated plants were grown inside the greenhouse for symptom observation and causal agent detection assays.

### RNA Extraction, RT-PCR, and Quantitative RT-PCR

Total RNA was extracted from leaf samples using TRNzol reagent (TIANGEN Biotechnology, Beijing, China). RT-PCR was performed using the PrimeScript™ One-Step RT-PCR Kit Ver.2 (TaKaRa Biotechnology, Dalian, China) as previously described (Liu et al., 2014). The primers used to amplify the four causal agents are listed in Supplementary Table 2. For RT-qPCR, the One Step TB Green® PrimeScript™ RT-PCR Kit was used as instructed (TaKaRa Biotechnology). Quantitative PCR was performed on the QuantStudio™ 7 Flex Real-Time PCR System (ABI) under the conditions: 42°C for 5 min; 95°C for 10 s; 40 cycles at 95°C for 5 s, and 60°C for 30 s; 95°C for 15 s; 60°C for 1 min, and 95°C for 15 s. The relative expression level of each assayed RNA was determined using the 2^−ΔΔ*C*(t)^ method (Livak and Schmittgen, 2001). For relative quantification of each RNA, the relative expression level of *N. benthamiana Actin* gene was used as the internal control. Three biological replicates with three technical replicates each were used in each treatment.

### Virus Purification and Transmission Electron Microscopy

Purification of TVDV virion was performed as described previously (Mo et al., 2010) with some modifications. Systemically infected leaf tissues (about 500 g) were harvested from the TVDV-YK inoculated *N. benthamiana* plants at 15 days post agro-infiltration, and homogenized in 1,000 ml of extraction buffer [0.1 M sodium phosphate buffer (PB), pH 6.0, 0.5% (*v*/*v*) β-Mercaptoethanol, 0.5% (*v*/*v*) cellulase (Solarbio, China)]. The homogenate was stirred at 25°C for 5 h and then emulsified after addition of a mixture of chloroform and 1-butanol (1:1, *v*/*v*). The emulsified sample was centrifuged in the Beckman JA10 rotor at 8,000 rpm for 15 min at 15°C. The upper aqueous phase was collected, mixed with Triton X-100 till 1% (*v*/*v*), and stirred gently for 30 min. After addition of 6% PEG6000 (*w*/*v*) and 0.3 M NaCl, the mixture was stirred again for 1 h at 4°C followed by storage at 4°C for 8 h. The sample was centrifuged at 10,000 *g* for 30 min at 4°C and the pellet was resuspended in 50 ml of storage buffer (0.1 M PB, pH 7.0) followed by 8 h storage at 4°C. The resuspended pellet solution was centrifuged at 5,000 rpm for 20 min and the supernatant was transferred onto a 30% sucrose cushion followed by 4 h ultracentrifugation at 40,000 rpm and 4°C in the Beckman Ti70 rotor. The pellet was resuspended in 1 ml of storage buffer, added on a linearized 10–40% (*w*/*v*) sucrose gradient in 0.1 M PB, pH 7, and centrifuged for 2.5 h at 32,000 rpm in a Beckman SW41 rotor. The sucrose gradient was fractionated (0.2 ml each) and the virion-containing fractions were confirmed through RT-PCR followed by 1.5 h ultracentrifugation in a Beckman Ti 90 rotor at 60,000 rpm and 4°C. The final pellet was resuspended in 0.2 ml storage buffer and used for Transmission Electron Microscopy (TEM) at the Yunnan Academy of Agricultural Sciences, Kunming, China. RNA and coat protein of the purified virion were confirmed through Western blot assay and RT-PCR.

### Virion Infectivity Assay

The purified TVDV-YK virion was mixed with a 10% sucrose solution at a ratio of 1:4 (*v*/*v*) and used to feed, through stretched Parafilm membranes, to non-viruliferous *M. persicae* for 10 h as described (D’Arcy et al., 1983). Fifteen aphids were transferred onto a healthy *N. benthamiana* plant (three plants total) and allowed them to feed on the plants for 5 days. The aphids were eliminated through pesticide application and the plants were analyzed for TVDV-YK systemic infection at about 2 weeks postaphid inoculation through RT-PCR.

### Western Blot Analysis

The purified TVDV-YK virion sample was heated for 5 min in a 95°C water bath and then analyzed in 12% sodium dodecyl sulfate–polyacrylamide gels (SDS-PAGE) through electrophoresis. After transferring protein to a PVDF membrane, the membrane was probed with a rabbit anti-TVDV CP polyclonal antibody followed by an HRP-conjugated goat anti-rabbit IgG antibody (Sigma-Aldrich, St. Louis, MO, Unites States). The detection signal was visualized using an ECL substrate kit as instructed (Thermo Fisher Scientific, Waltham, MA, United States).

### Aphid Acquisition and Transmission Assay

A *M. persicae* colony was established from a single female collected from a field tobacco plant in 2019. The progeny aphids were maintained on healthy *N. benthamiana* or tobacco cv. K326 plants inside plastic cages in a growth chamber maintained at 22°C, 60% relative humidity, and 16/8 h (day/night) photoperiod. The aphid acquisition efficiencies of the four TBTD causal agents were determined as described by Fereres et al. (1993). Briefly, 60 non-viruliferous young apterous adults were starved for 3 h and then maintained on the tobacco plants inoculated with different infectious clones. After 24 h, total RNA was extracted from individual aphids using TransZol Up reagent (TransGen, Beijing, China) followed by RT-PCR detection. For each treatment, 20 aphids were tested and the experiment was repeated three times. For aphid transmission assays, aphids were collected after 24-h acquisition and transferred onto healthy tobacco K326 plants (20 aphids per plant). After 5 day feeding, the aphids were eliminated through pesticide application. Systemic virus infection in the assayed plants was determined at about 2 weeks postaphid inoculation through RT-PCR using specific primers (Supplementary Table 2). This experiment was repeated three times.

## Results

### Sequencing, Sequence Alignment and Phylogenetic Analysis of TBTD Causal Agents

**Figure 1:**
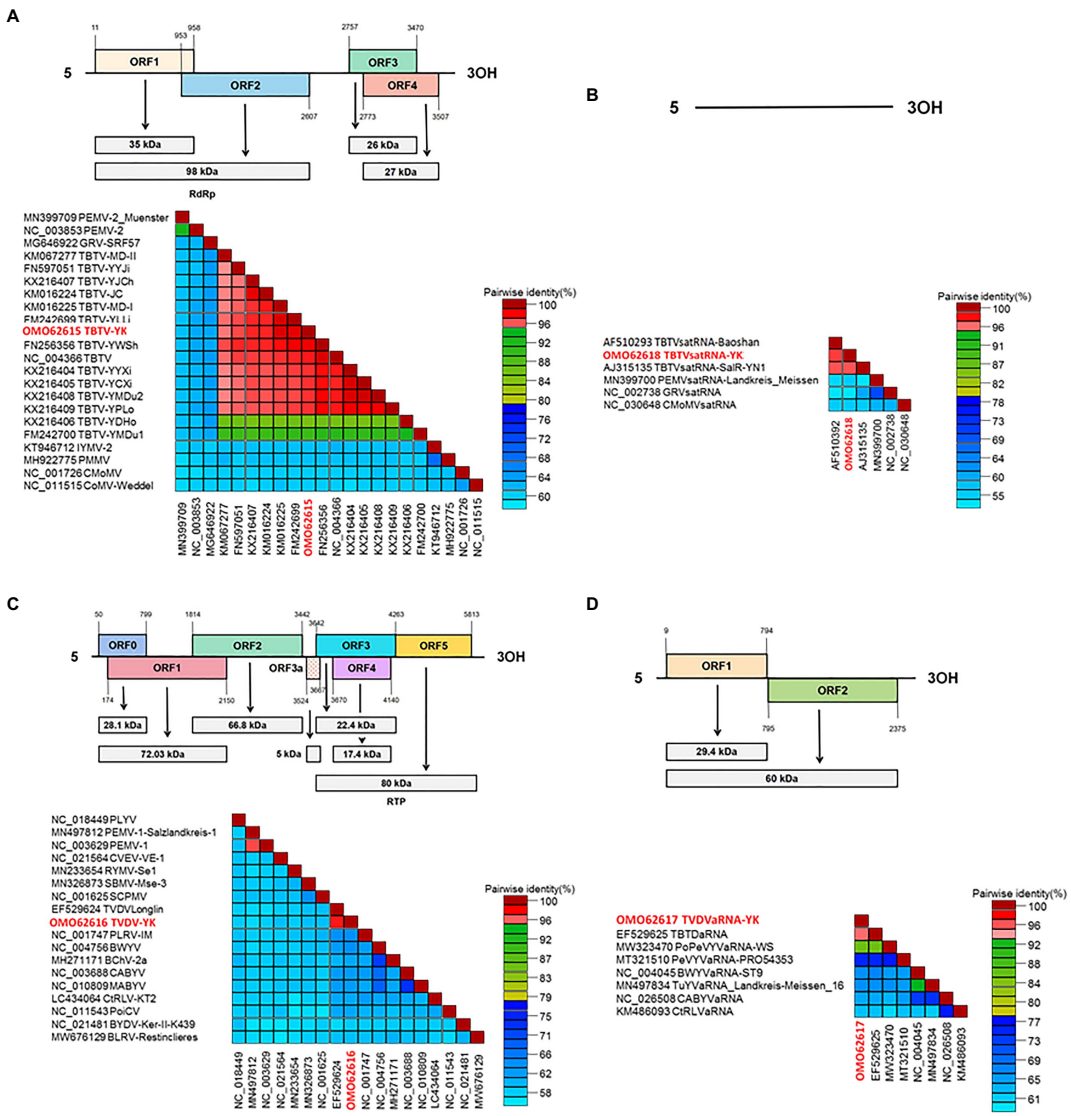
Genome organization of TBTV-YK, TVDV-YK, TBTVsatRNA-YK, and TVDVaRNA-YK, and the pairwise identity matrices of complete nucleotide (nt) sequences generated using the Sequence Demarcation Tool version 1.2. **(A)** Genome structure of TBTV-YK and the pairwise identity matrix of TBTV-YK and other viruses in the genus *Umbravirus*, *Tombusvirus*, and *Luteovirus* in the family *Tombusviridae*. **(B)** Genome structure of TBTVsatRNA-YK and the pairwise identity matrix of TBTVsatRNA-YK and other satellite RNAs associated with the viruses in the genus *Umbravirus*. **(C)** Genome structure of TVDV-YK and the pairwise identity matrix of TVDV-YK and other viruses in the genus *Polemovirus*, *Polerovirus*, *Sobemovirus*, and *Enamovirus* in the family *Solemoviridae* and the viruses in the family *Tombusviridae*. **(D)** Genome structure of TVDVaRNA-YK and the pairwise identity matrix of TVDVaRNA-YK and other Tombusvirus-like associated RNAs.

Based on our sequencing results, the complete sequence of TBTV-YK contains 4,152 nucleotides (nt) and has four ORFs (Figure 1A). Pairwise alignment result showed that TBTV-YK shares the highest nt sequence similarity (99.52%) with that of TBTV-MD-I, and 59.85–63.91% nt sequence similarities with other reported umbraviruses. In addition, it shares 46.29–49.90% nt sequence similarities with some viruses in the genus *Tombsuvrus* or *Luteovirus*, family *Tombusviridae*. The TBTVsatRNA-YK sequence contains 824 nts (without a single ORF) and shares the highest nt sequence similarity (89.59%) with that of TBTVsatRNA-SalR-YN1 (Figure 1B). The TVDV-YK sequence contains 5,918 nts and has seven ORFs, similar to other known poleroviruses. TVDV-YK shears only 37.51–51.77% nt sequence similarities with other reported members in the genus *Polerovirus*, *Polemovirus*, *Sobemovirus* or *Enamovirus*, family *Solemoviridae*, and 53.75–54.18% nt sequence similarities with that of BLRV-Restinclieres and BYDV Ker-II-K439, which were previously grouped in the family *Luteoviridae* (Figure 1C). The TVDVaRNA-YK sequence contains 2,971 nts and shears 95.32% nt sequence similarity with TBTDaRNA. TVDVaRNA-YK is predicted to have two ORFs (i.e., ORF1a and the readthrough protein ORF1b), characteristic of tlaRNAs (Figure 1D).

**Figure 2:**
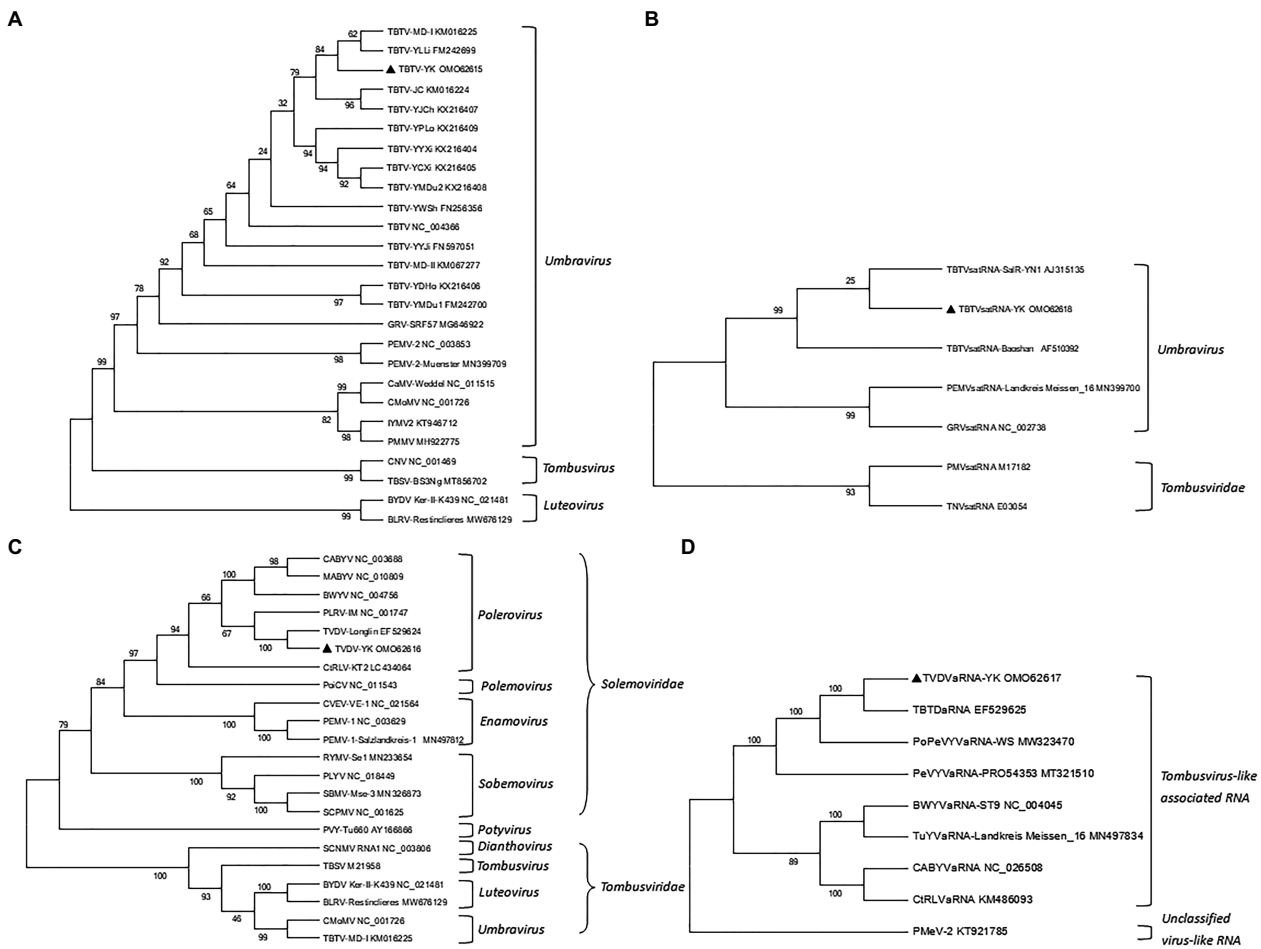
Neighbor-joining phylogenetic trees constructed using the complete nucleotide (nt) sequences of TBTV-YK, TBTVsatRNA-YK, TVDV-YK, TVDVaRNA-YK, and other related viruses. **(A)** A phylogenetic tree constructed using the nt sequences of TBTV-YK and other viruses in the genus *Umbravirus*, *Tombusvirus*, and *Luteovirus*. **(B)** A phylogenetic tree constructed using the nt sequences of TBTVsatRNA-YK and other satellite RNAs in the genus *Umbravirus*. **(C)** A phylogenetic tree constructed using the nt sequences of TVDV-YK and other viruses in the genus *Polemovirus*, *Polerovirus*, *Sobemovirus*, and *Enamovirus* in the family *Solemoviridae* or in the family *Tombusviridae*. **(D)** A phylogenetic tree constructed using the nt sequences of TVDVaRNA-YK and other Tombusvirus-like associated RNAs.

To investigate the phylogenetic relationships among the TBTV, TVDV, TBTVsatRNA or TVDVaRNA isolates, we constructed four phylogenetic trees using the nt sequences of TBTV-YK, TBTVsatRNA-YK, TVDV-YK, TVDVaRNA-YK, and those retrieved from the GenBank using the neighbor-joining method in the MEGA 7.0 software with 1,000 bootstrap replications (Figure 2). The resulting phylogenetic trees showed that TBTV-YK is closely related to TBTV-YLLi and TBTV-MD-I (Figure 2A), while TBTVsatRNA-YK is closely related to TBTVsatRNA-SalR-YN1 (Figure 2B). TVDV-YK is closely related to TVDV-Longlin (Figure 2C), and TVDVaRNA-YK is closely related to TBTDaRNA (Figure 2D).

### TBTV-YK, TVDV-YK, TVDVaRNA-YK, and/or TBTVsatRNA-YK Induced TBTD Symptoms in Tobacco K326 Plants

**Figure 3:**
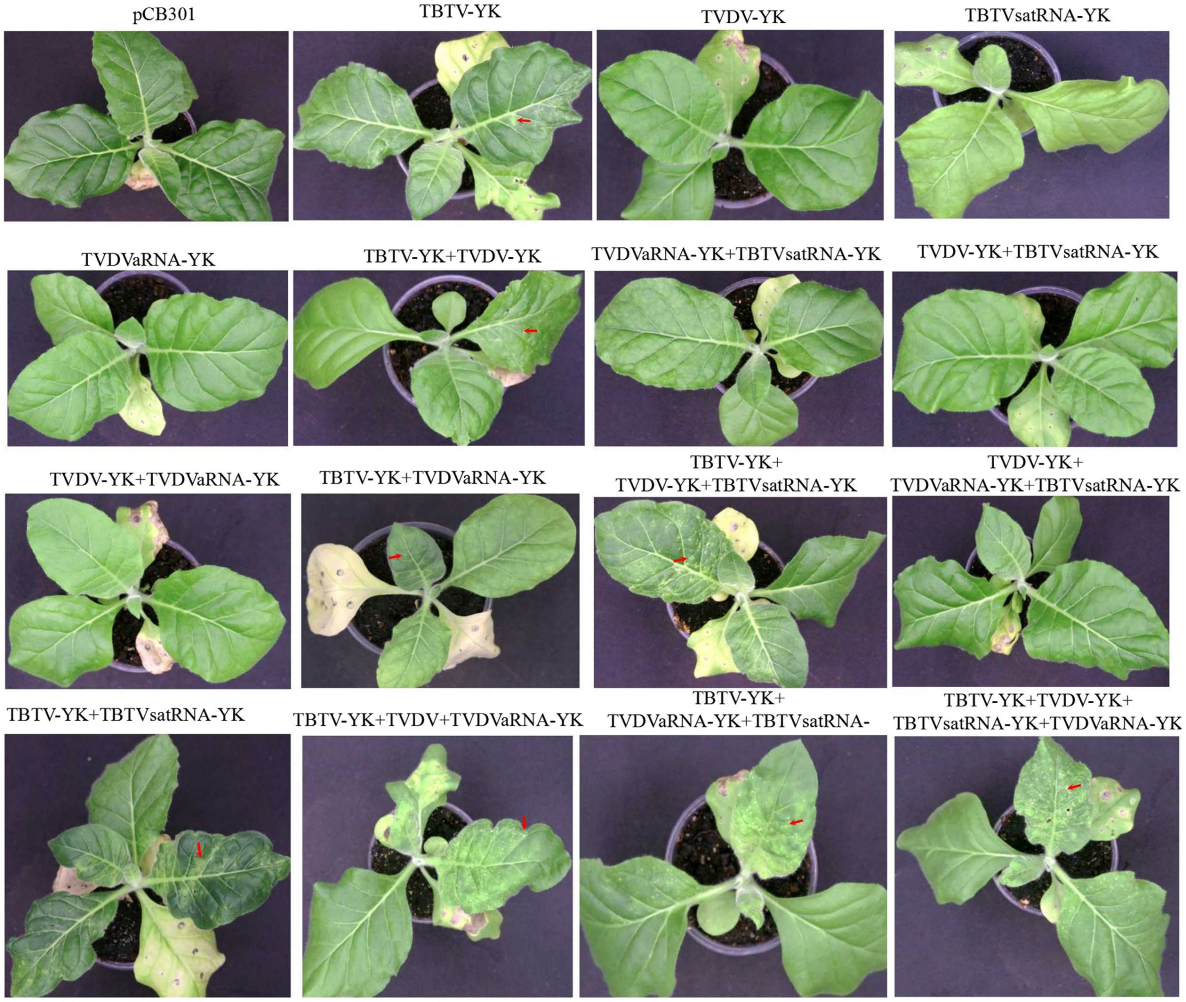
Symptoms on the tobacco K326 plants inoculated with TBTV-YK, TVDV-YK, TBTVsatRNA-YK, and TVDVaRNA-YK, singly or in various combinations, through agro-infiltration. Arrows indicate necrotic spots in the systemic leaves. The plants were photographed at 14 days postinoculation. The plant agro-infiltrated with pCB301 (an empty expression vector) was used as a control.

**Figure 4:**
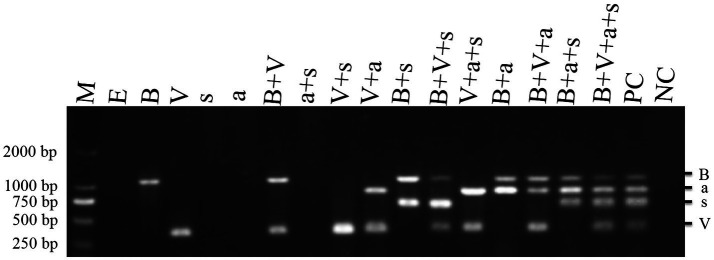
RT-PCR detections of TBTD causal agents in the assayed tobacco K326 plants at 14 dpi. Lanes M, DL 2000 DNA Marker; lane E, plants inoculated with pCB301; lane B, plants inoculated with TBTV-YK; lane V, plants inoculated with TVDV-YK; lane s, plants inoculated with TBTVsatRNA-YK; lane a, plants inoculated with TVDVaRNA-YK; lane B+V, plants inoculated with TVDV-YK+TBTV-YK; lane a+s, plants inoculated with TVDVaRNA-YK+TBTVsatRNA-YK; lane V+s, plants inoculated with TVDV-YK+TBTVsatRNA-YK; lane B+s, plants inoculated with TBTV-YK+TBTVsatRNA-YK; lane V+a, plants inoculated with TVDV-YK+TVDVaRNA-YK; lane V+B+s, plants inoculated with TVDV-YK+TBTV-YK+TBTVsatRNA-YK; lane V+a+s, plants inoculated with TVDV-YK+TVDVaRNA-YK+TBTVsatRNA-YK; lane B+a+s, plants inoculated with TBTV-YK+TVDVaRNA-YK+TBTVsatRNA-YK; lane V+B+a+s, plants inoculated with TVDV-YK+TBTV-YK+TVDVaRNA-YK+TBTVsatRNA-YK. PC, Positive control; NC, negative control using healthy tobacco K326 plant samples.

To determine the pathogenicity of the four TBTD causal agents in tobacco plants, we first inoculated *N. benthamiana* and tobacco K326 plants with TVDV-YK, TBTV-YK, TBTVsatRNA-YK, and TVDVaRNA-YK, individually or in various combinations, through agro-infiltration. By 7 dpi, the tobacco K326 plants co-inoculated with the four causal agents showed necrosis in their systemic young leaves (Figure 3) followed by leaf distortion and plant stunting by 30 dpi and in Supplementary Figure 1 showed the symptoms of 101 dpi. In this study, the plants inoculated with TBTV-YK alone also showed necrosis in their systemic young leaves by 7 dpi, while the plants inoculated with TVDV-YK, TBTVsatRNA-YK or TVDVaRNA-YK alone did not show clear virus-like symptoms in their systemic leaves (Figure 3). Results of RT-PCR showed that TBTV-YK or TVDV-YK RNA had accumulated in the systemic leaves of the TBTV-YK or TVDV-YK inoculated plants (Figure 4). However, TBTVsatRNA-YK or TVDVaRNA-YK RNA was not detected in the systemic leaves of the TBTVsatRNA-YK or TVDVaRNA-YK inoculated plants. In the tobacco K326 plants co-inoculated with TBTVsatRNA-YK and TVDVaRNA-YK, both RNAs were also not detected in their systemic leaves through RT-PCR.

**Figure 5:**
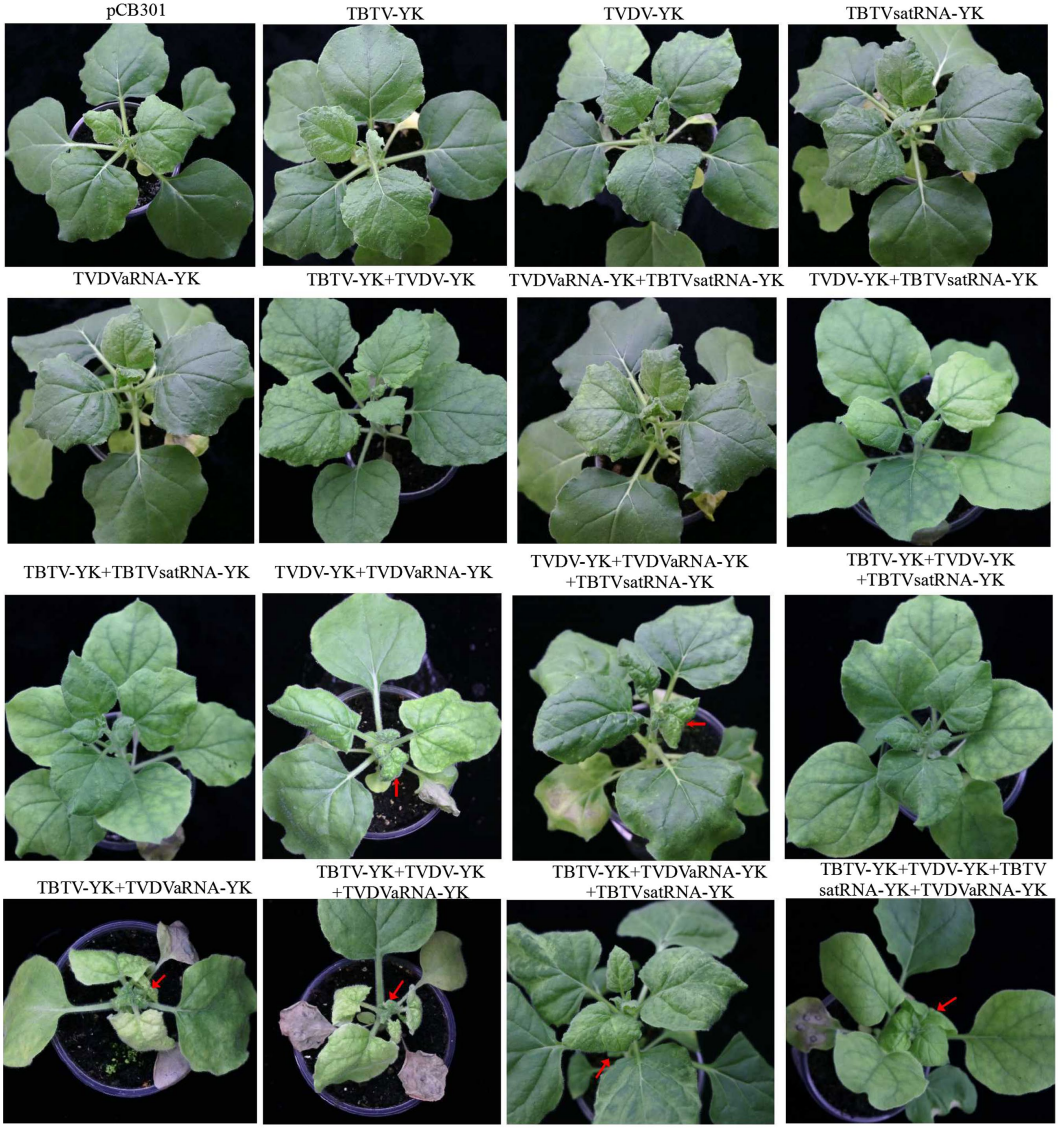
Symptoms on the *Nicotiana benthamiana* plants inoculated with TBTV-YK, TVDV-YK, TBTVsatRNA-YK, and TVDVaRNA-YK, singly or in various combinations, through agro-infiltration. Arrows indicate curly top-like symptoms. The plants were photographed at 16 days postinoculation. The plant agro-infiltrated with pCB301 (an empty expression vector) was used as a control.

**Figure 6:**
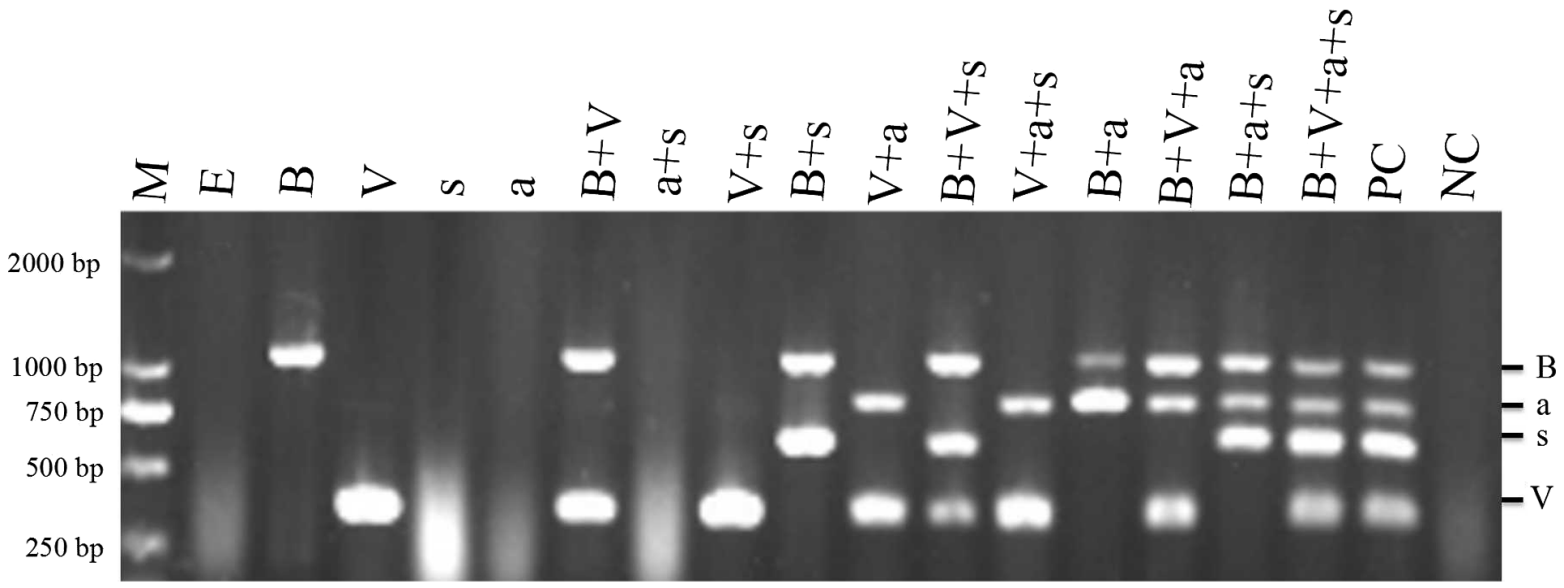
RT-PCR detections of TBTD causal agents in the assayed *Nicotiana benthamiana* plants at 16 dpi. Lane M, DL 2000 DNA Marker; lane E, plants inoculated with pCB301; lane B, plants inoculated with TBTV-YK; lane V, plants inoculated with TVDV-YK, lane s, plants inoculated with TBTVsatRNA-YK; lane a, plants inoculated with TVDVaRNA-YK; lane B+V, plants inoculated with TVDV-YK+TBTV-YK, lane a+s, plants inoculated with TVDVaRNA-YK+TBTVsatRNA-YK; lane V+s, plants inoculated with TVDV-YK+TBTVsatRNA-YK; lane B+s, plants inoculated with TBTV-YK+TBTVsatRNA-YK; lane V+a, plants inoculated with TVDV-YK+TVDVaRNA-YK, lane V+B+s, plants inoculated with TVDV-YK+TBTV-YK+TBTVsatRNA-YK, lane V+a+s, plants inoculated with TVDV-YK+TVDVaRNA-YK+TBTVsatRNA-YK; lane B+a+s, plants inoculated with TBTV-YK+TVDVaRNA-YK+TBTVsatRNA-YK; lane V+B+a+s, plants inoculated with TVDV-YK+TBTV-YK+TVDVaRNA-YK+TBTVsatRNA-YK. PC, positive control; NC: negative control using healthy *Nicotiana benthamiana* plant samples.

When *N. benthamiana* plants were co-inoculated with TVDV-YK, TBTV-YK, TBTVsatRNA-YK, and TVDVaRNA-YK, the inoculated plants developed leaf curling symptoms by 14 dpi. By 16 dpi, the co-inoculated plants showed strong leaf distortion symptoms (Figure 5), and by 42 dpi, the co-inoculated plants showed strong leaf curling and yellowing, malformed flowers, and plant stunting (Supplementary Figure 2). In this study, the *N. benthamiana* plants inoculated with TBTV-YK or TVDV-YK alone did not show any obvious symptoms on their systemic leaves by 16 dpi (Figure 5). By 74 dpi, these TBTV-YK-inoculated plants showed smaller leaves and plant stunting (Supplementary Figure 3), while the plants inoculated with TVDV-YK alone did not show clear virus-like symptoms in their systemic leaves. Result of RT-PCR showed that TBTV-YK or TVDV-YK RNA had indeed accumulated in the systemic leaves of the TBTV-YK or TVDV-YK inoculated *N. benthamiana* plants (Figure 6). As expected, the plants inoculated with TBTVsatRNA-YK or TVDVaRNA-YK alone showed no obvious disease symptoms and had not accumulated TBTVsatRNA-YK or TVDVaRNA-YK RNA in their systemic leaves.

### TVDVaRNA Enhances TBTV Induced Disease Symptoms in *Nicotiana benthamiana*

**Figure 7:**
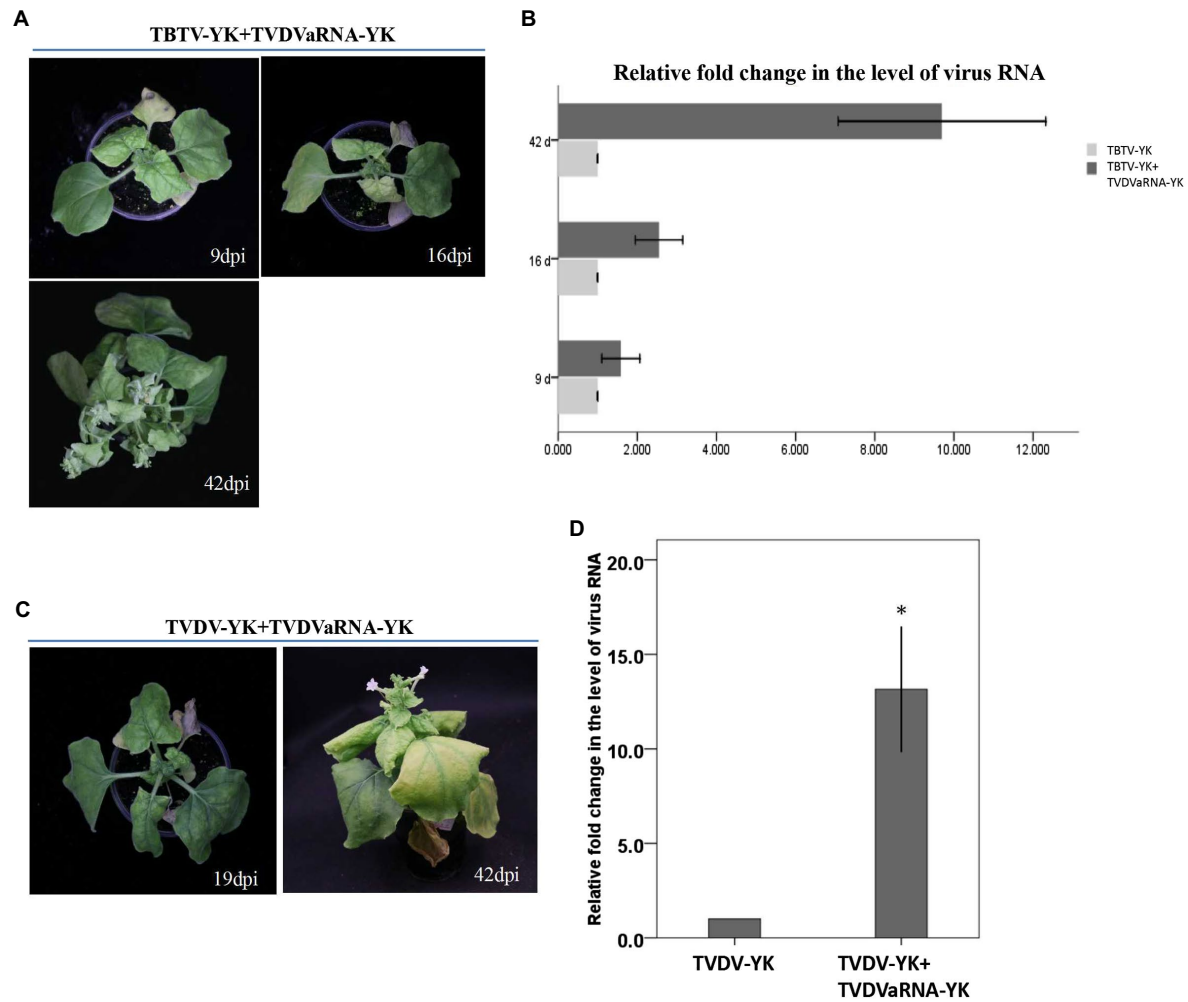
Symptoms caused by TBTV-YK and TVDV-YK in *Nicotiana benthamiana* co-infected with TVDVaRNA-YK, and quantitative RT-PCR analyses of TBTV-YK and TVDV-YK RNA accumulations in the systemic leaves of *Nicotiana benthamiana* plants inoculated with TBTV-YK, TVDV-YK alone or with TVDVaRNA-YK. **(A)** Phenotypes of *Nicotiana benthamiana* plants inoculated with TBTV-YK+TVDVaRNA-YK *via* agro-infiltration. The plants were photographed at 9, 16, and 42 days postinoculation (dpi). **(B)** Quantitative RT-PCR analyses of TBTV-YK RNA accumulations in the systemic leaves of *Nicotiana benthamiana* plants inoculated with TBTV-YK alone or with TBTV-YK+TVDVaRNA-YK. The means ± SE were calculated using the data from three individual plants harvested at 9, 16, and 42 dpi. **(C)** Phenotypes of *Nicotiana benthamiana* plants inoculated with TVDV-YK+TVDVaRNA-YK *via* agro-infiltration. The plants were photographed at 19 and 42 dpi. **(D)** Quantitative RT-PCR analyses of TVDV-YK RNA accumulations in the systemic leaves of *Nicotiana benthamiana* plants inoculated with TVDV-YK alone or with TVDV-YK+TVDVaRNA-YK. The means ± SE were calculated using the data from three individual plants harvested at 19 dpi.

To investigate the synergistic effect of TVDVaRNA-YK in co-infected plants, we co-inoculated *N. benthamiana* and tobacco K326 plants with TBTV-YK and TVDVaRNA-YK or TVDV-YK and TVDVaRNA-YK through agro-infiltration. Both TBTV-YK and TVDVaRNA-YK (TBTV-YK+TVDVaRNA-YK) and TVDV-YK and TVDVaRNA-YK (TVDV-YK+TVDVaRNA-YK) co-inoculated *N. benthamiana* plants showed strong leaf curling and yellowing symptoms in their systemic leaves and plant stunting (Figure 7). In contrast, the TBTV-YK+TVDVaRNA-YK and TVDV-YK+TVDVaRNA-YK co-inoculated tobacco plants did not show clear plant stunting symptoms (Figure 3). The time course study result showed that the TBTV-YK+TVDVaRNA-YK co-inoculated *N. benthamiana* plants started to show leaf curling symptoms by 7 dpi. By 42 dpi, the disease symptoms became leaf chlorosis and strong plant stunting (i.e., bushy top; Figure 7A). The TVDV-YK+TVDVaRNA-YK co-inoculated plants started to show leaf curling symptoms by 14 dpi, and by 42 dpi, the symptoms became leaf yellowing and plant stunting (Figure 7C). The result of RT-PCR showed that TBTV-YK RNA had accumulated in the systemic leaves of the TBTV-YK inoculated or the TBTV-YK+TVDVaRNA co-inoculated *N. benthamiana* plants (Figure 6). Similarly, TVDV-YK RNA had accumulated in the systemic leaves of the *N. benthamiana* plants inoculated with TVDV-YK or TVDV-YK+TVDVaRNA-YK. The RT-PCR results also showed that TVDVaRNA-YK RNA had accumulated in the systemic leaves of the TBTV-YK+TVDVaRNA-YK or TVDV-YK+TVDVaRNA-YK co-inoculated plants. In addition, the result of RT-qPCR showed that the accumulation levels of TBTV-YK RNA in the TBTV-YK+TVDVaRNA-YK co-inoculated plants increased by 1.58-, 2.55-, and 9.7-fold, compared with that in the TBTV-YK inoculated plants, at 9, 16, and 42 dpi, respectively (Figure 7B). Similarly, the accumulation level of TVDV-YK RNA in the TVDV-YK+TVDVaRNA-YK co-inoculated plants increased by 14.4 fold, compared with that in the TVDV-YK inoculated plants, at 19 dpi (Figure 7D).

### Role of TVDV-YK in Aphid Transmission of TBTD Causal Agents

**Figure 8:**
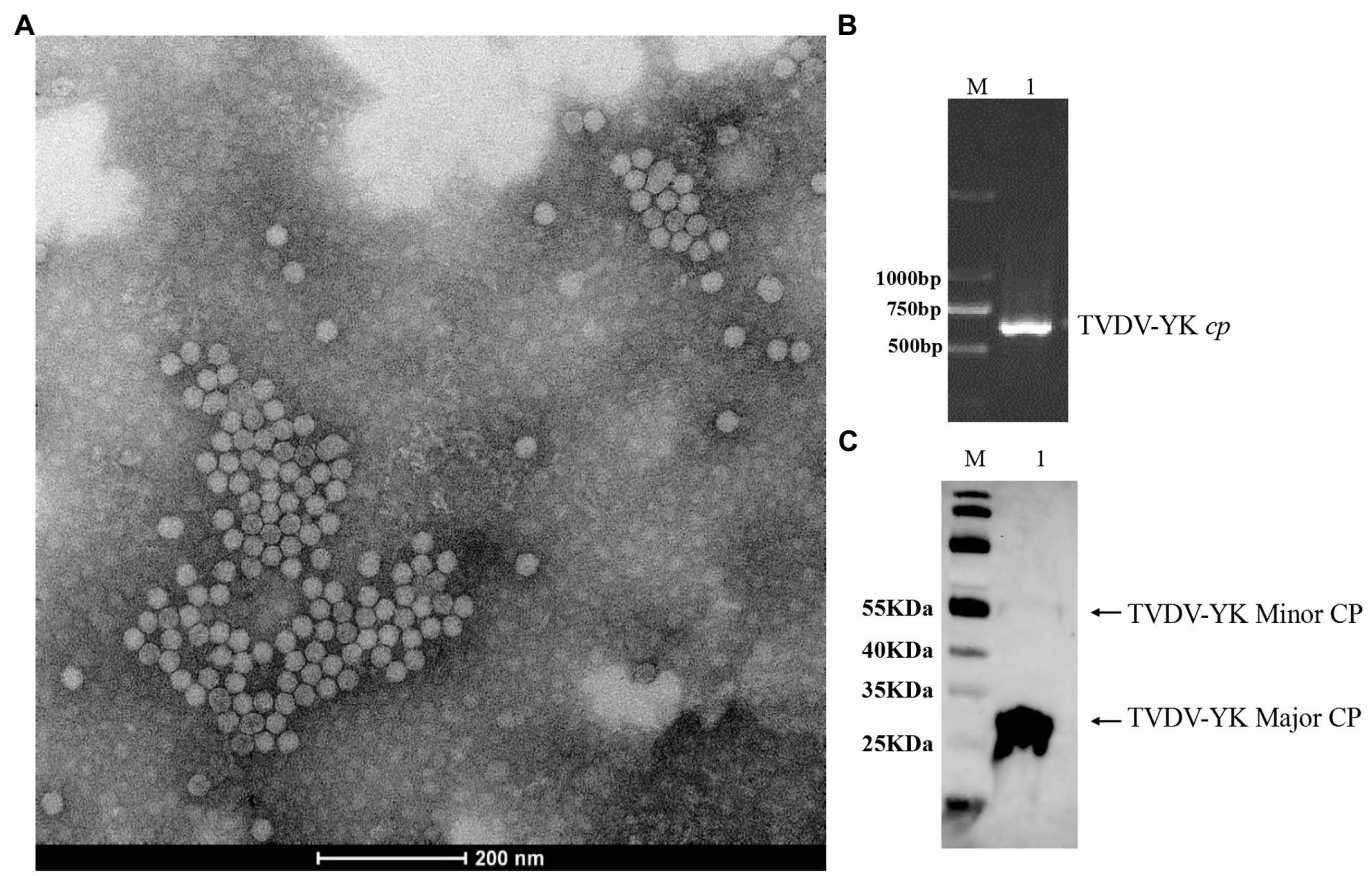
Virions purified from *Nicotiana benthamiana* leaves infected by the TVDV-YK infectious clones, observed by TEM, and detected by RT-PCR and western blotting. **(A)** An electron microscopic image of TVDV-YK virions purified from TVDV-YK infected *Nicotiana benthamiana* leaves. The virions were negatively stained with 1% phosphotungstic acid and photographed under an electron microscope. Bar, 200 nm. **(B)** RT-PCR products from purified TVDV-YK virions. Lane M, DL 2000 DNA Marker. **(C)** Western blot detection of coat protein (CP) from the purified TVDV-YK virions using a polyclonal antibody specific for TVDV CP. Lanes M: 26616 protein ladder (Thermo Fisher Scientific, Waltham, MA, Unites States).

To investigate the infectivity of TVDV-YK, we first purified TVDV-YK virions from the systemic leaves of the TVDV-YK inoculated plants and examined them under an electron microscope. The result showed that TVDV-YK virions are about 28 nm icosahedral particles (Figure 8A). The presence of TVDV-YK RNA and coat protein in the purified TVDV-YK virion sample was confirmed by RT-PCR using the *cp* gene primers and Western blot assay using an antiserum specific for TVDV-YK CP, respectively (Figures 8B,C). Inoculation of purified TVDV-YK virions to *N. benthamiana* plants through *M. persicae*, the main transmission vector of TVDV in field, followed by RT-PCR showed that the purified TVDV-YK was capable of infecting and spread systemically in *N. benthamiana* plants (Supplementary Table 3).

To explore the roles of TBTV-YK, TVDV-YK, and TBTVsatRNA-YK in aphid transmission. *M. persicae* was allowed to feed on the *N. benthamiana* plants infected with one, two, or three causal agents. After acquisition seeding, the aphids were used to inoculate healthy tobacco K326 plants. Analysis of aphids through RT-PCR showed that none of the 60 aphids (three experiments with 20 aphids each) fed on the plants infected with TBTV-YK alone was viruliferous. In contrast, 35 aphids (58.3%) fed on the plants infected with TVDV-YK were viruliferous. In addition, 10 aphids (16.7%) fed on the plants co-infected with TBTV-YK and TVDV-YK, and 22 aphids (36.7%) fed on the plants co-infected with TBTV-YK, TVDV-YK, and TBTVsatRNA-YK were viruliferous (Table 1), suggesting that the presence of TBTVsatRNA-YK can increase the efficiency of aphid acquisition of TBTV-YK and TVDV-YK. In this study, we also tested the efficiency of aphid transmission of different causal agents to tobacco K326 plants (Table 2). The RT-PCR results showed that *M. persicae* was unable to transmit TBTV-YK from the TBTV-YK infected *N. benthamiana* plants to tobacco K326 plants. When aphids were allowed to feed on the TBTV-YK+TVDV-YK co-infected *N. benthamiana* plants and then on the tobacco K326 plants, about 21.7% of the inoculated tobacco K326 plants became co-infected with the two causal agents. When aphids were allowed to feed on the TBTV-YK+TVDV-YK+TBTVsatRNA-YK co-infected *N. benthamiana* plants and then on the tobacco K326 plants, about 35% of the inoculated tobacco K326 plants became co-infected with the three causal agents, suggesting that the presence of TBTVsatRNA-YK can increase the efficiency of aphid transmission of TBTV-YK and TVDV-YK to tobacco plants.

**Table 1:** Acquisition efficiencies of TBTV-YK, TVDV-YK, and TBTVsatRNA-YK of TBTD by *Myzus persicae*.

Treatments	Acquisition (No. of aphids acquired virus/feeding)						
	TBTV-YK	TVDV-YK	TBTVsatRNA-YK	TBTV-YK+TVDV-YK	TVDV-YK+TBTVsatRNA-YK	TBTV-YK+TBTVsatRNA-YK	TBTV-YK+TVDV-YK+TBTVsatRNA-YK
TBTV-YK	0/20	–	–	–	–	–	–
	0/20	–	–	–	–	–	–
	0/20	–	–	–	–	–	–
TVDV-YK	–	9/20	–	–	–	–	–
	–	12/20	–	–	–	–	–
	–	14/20	–	–	–	–	–
TBTV-YK+TVDV-YK	1/20	9/20	–	4/20	–	–	–
	0/20	12/20	–	3/20	–	–	–
	3/20	5/20	–	3/20	–	–	–
TBTV-YK+TVDV-YK+TBTVsatRNA-YK	0/20	1/20	1/20	0/20	7/20	5/20	5/20
	0/20	1/20	8/20	1/20	2/20	1/20	4/20
	0/20	0/20	0/20	0/20	6/20	0/20	13/20

*Non-viruliferous aphids were used to feed *Nicotiana benthamiana* plant infected with TVDV-YK, TBTV-YK, TVDV-YK+TBTV-YK, and TBTV-YK+TVDV-YK+TBTVsatRNA-YK. After 24 h, the one-step RT-PCR was used to detect an aphid acquired virus. “–” represents no detection*.

**Table 2:** Transmission efficiencies of TBTV-YK, TVDV-YK, and TBTVsatRNA-YK of TBTD by *Myzus persicae*.

Treatments	Transmission (No. of plants infected/transmission)						
	TBTV-YK	TVDV-YK	TBTV satRNA-YK	TBTV-YK+TVDV-YK	TVDV-YK+TBTVsatRNA-YK	TBTV-YK+TBTV satRNA-YK	TBTV-YK+TVDV-YK+TBTVsatRNA-YK
TBTV-YK	0/20	–	–	–	–	–	–
	0/20	–	–	–	–	–	–
	0/20	–	–	–	–	–	–
TVDV-YK	–	7/22	–	–	–	–	–
	–	5/16	–	–	–	–	–
	–	16/22	–	–	–	–	–
TBTV-YK+TVDV-YK	4/30	9/30	–	8/30	–	–	–
	0/22	0/22	–	2/22	–	–	–
	0/8	5/8	–	3/8	–	–	–
TBTV-YK+TVDV-YK+TBTVsatRNA-YK	0/9	1/9	0/9	0/9	0/9	2/9	6/9
	1/32	5/32	0/32	1/32	7/32	1/32	9/32
	0/19	3/19	5/19	1/19	2/19	1/19	6/19

*The RT-PCR was used to detect *tobacco K326* plants after aphid inoculation at 14 days. “–” represents no detection*.

## Discussion

In this study, we have determined the full-length nucleotide sequences of TBTV-YK, TVDV-YK, TBTVsatRNA-YK, and TVDVaRNA-YK in tobacco plants collected from tobacco fields in the Kunming City, Yunnan Province, China, through high throughput sequencing (HTS) and RT-PCR. Four infectious clones, representing TBTV-YK, TVDV-YK, TBTVsatRNA-YK, and TVDVaRNA-YK, were constructed. Through agro-infiltration of these infectious clones, separately or in various combinations, into leaves of *N. benthamiana* and tobacco K326 plants, the roles of individual causal agents on induction of bushy top disease symptoms were determined. In this study, we have also investigated the roles of individual causal agents in aphid transmission. These results should benefit the establishment of efficient TBTD management strategies and TBTD resistant tobacco breeding programs.

It was reported that the severity of virus infection in plants is host plant species dependent. For example, the *N. clevelandii* or *N. benthamiana* plants inoculated with PEMV alone showed strong mosaic and leaf curling symptoms, but the *N. tabacum* plants inoculated with PEMV alone showed symptomless infection (Motoyoshi and Hull, 1974; Demler et al., 1994). In this study, the TBTV-YK inoculated tobacco K326 plants showed necrosis in their young systemic leaves by 7 dpi and necrosis, reduced leaf size, and shorter internodes by 45 dpi. In contrast, the TBTV-YK inoculated *N. benthamiana* plants did not show obvious disease symptoms by 7 dpi and showed only reduced leaf size, clumping branches, and malformed flowers by 45 dpi. This finding indicates that these two host plant species have different anti-TBTV infection mechanisms, and thus provides an interesting avenue to elucidate the molecular mechanism controlling the arms races between TBTV and different plants.

Although satRNAs are known to be dispensable for helper virus replication, they can influence helper virus RNA accumulation and disease symptom induction in infected plants (Collmer and Howell, 1992). In this study, we have determined that the tobacco K326 plants co-inoculated with TBTV-YK and TBTVsatRNA-YK showed more severe necrosis in leaves compared to the plants inoculated with TBTV-YK alone. Therefore, we conclude that TBTVsatRNA-YK can promote TBTV-YK accumulation and symptom induction in tobacco K326 plants. This observation agrees with a previous report that GRV satRNA is responsible for the induction of rosette disease symptoms in groundnut plants and for the production of yellow blotch symptoms in *N. benthamiana* plants (Taliansky and Robinson, 1997). It is noteworthy that in that report, the presence of satRNA YB3b did not affect the accumulation of GRV genomic and subgenomic RNA. Taliansky and Robinson (1997) reported that trans-acting untranslated elements of groundnut rosette virus satellite RNA are involved in replication and symptom induction. The result of phylogenetic analysis indicated that TBTVsatRNA-YK is close to GRV satRNA. Therefore, we speculate that TBTVsatRNA-YK may contain some unidentified trans-acting untranslated element(s) that can regulate TBTV-YK replication, or interfere with host defense pathways to promote the expressions of TBTV-YK proteins.

Several tlaRNAs have been reported to enhance polerovirus accumulations in infected plants and to intensify disease symptoms (Sanger et al., 1994; Mo et al., 2015; Yoshida, 2020; Peng et al., 2021). Through sequencing and phylogenetic analyses, we have determined that TVDVaRNA-YK is evolutionary closely related to TBTDaRNA-YK. A previous report had indicated that TVDV can traffic TBTDaRNA systemically in plants, while TBTDaRNA can enhance the TVDV infection-induced disease symptoms in *N. benthamiana* (Mo et al., 2015). In this study, we have found that TVDV-YK can also traffic TVDVaRNA-YK systemically in co-infected plants. On the other hand, TVDVaRNA-YK can intensify the TVDV-YK infection-induced disease symptoms as well as TVDV-YK accumulation. Furthermore, we have found that TBTV-YK can also facilitate TVDVaRNA-YK systemic movement in the co-infected plants, while TVDVaRNA-YK can increase TBTV-YK accumulation. In a recent report, CtRLVaRNA was found to co-infect plants with CMoV through aphid transmission (Yoshida, 2020). Unfortunately, how CtRLVaRNA interact with CMoV during aphid transmission is not clear. Analysis of the TVDVaRNA-YK inoculated plants through RT-PCR showed that TVDVaRNA-YK alone was incapable of moving systemically in plants. Because umbravirus encoded proteins have been reported to stabilize and traffic heterologous RNA in plants (Ryabov et al., 1999, 2001), we speculate that the TBTV-YK encoded protein(s) may be capable of interacting with TVDVaRNA-YK to assist its systematic spread in plants. Previous field investigations have found that TVDVaRNA-YK could be detected together with TBTV-YK, but not TVDV-YK, in some field collected tobacco samples, further indicating the possibility of interaction between the umbravirus protein(s) and TVDVaRNA-YK. The result of phylogenetic analysis using conserved domains in RdRps indicated that tlaRNAs can be placed together in a monophyletic clade, together with the members in the family *Tombusviridae* (Campbell et al., 2019). Our sequence alignment results suggested that TVDVaRNA-YK is a tlaRNA, and TBTV-YK and TVDVaRNA-YK are evolutionarily related. We speculate that the interaction between the RdRps of the two viruses may cause an enhancement of disease symptoms in co-infected plants. Our results have also shown that TBTV-YK, but not TVDV-YK, can facilitate TBTVsatRNA-YK movement in infected plants, indicating that the support of satellite RNA spread in plants is helper virus-specific (Roossinck et al., 1992). This speculation is supported by a report that lucerne transient streak virus (LTSV) can support the replication of solanum nodiflorum mottle virus (SNMV) satRNA, while SNMV is unable to support the replication of LTSVsatRNA (Jones and Mayo, 1984).

TBTD causal agents can be spread to other crop species through aphid vectors (Li et al., 2018, 2021; Tan et al., 2021). To investigate the interactions among different TBTD causal agents during aphid transmission, we conducted assays using *N. benthamiana* plants infected with one or more causal agents as the infection sources and tobacco K326 plants as the recipients to determine the rate of aphid transmission. The results show that TBTV-YK and TVDV-YK can be co-acquired from the infected *N. benthamiana* plants and co-transmitted to tobacco K326 plants by *M. persicae*. When TBTVsatRNA-YK was also present in the co-infected *N. benthamiana* plants, all three causal agents can be more efficiently acquired by aphids and transmitted to tobacco K326 plants. Through RT-qPCR analyses, we have found that the accumulation level of TBTV-YK was increased due to the presence of TBTVsatRNA-YK. We have also found that in the presence of TBTVsatRNA-YK, the aphid acquisition and transmission efficiency of TBTV and TVDV were increased. It is known that viruses in the family *Luteoviridae* are transmitted by aphids in the form of virus particles (Gray et al., 2014; Byrne et al., 2019). Robinson et al. (1999) had reported that GRVsatRNA could increase the encapsidation of GRV RNA, leading to an increased GRV virions and aphid transmission. ST9a RNA has also been shown to promote the encapsidation of satRPV RNA by BWYV capsid protein (Passmore et al., 1993). Whether TBTVsatRNA-YK can also increase TBTV encapsidation will be further investigated in future experiments.

## Data Availability Statement

The datasets presented in this study can be found in online repositories. The names of the repository/repositories and accession number(s) can be found at: https://www.ncbi.nlm.nih.gov/genbank/, OMO62615; https://www.ncbi.nlm.nih.gov/genbank/, OMO62616; https://www.ncbi.nlm.nih.gov/genbank/, OMO62617; and https://www.ncbi.nlm.nih.gov/genbank/, OMO62618.

## Author Contributions

XC, HL, JZ, HC, and FL conceived and designed the experiments. XC, HL, JZ, and KL performed the experiments. YM and FX contributed to construct the full-length infectious clones. YY and PL contributed to reagents, materials, and analysis tools. JY contributed to virus purification. XC, JZ, TW, YX, and FL wrote the manuscript. All authors contributed to the article and approved the submitted version.

## Funding

This project was financially supported by the National Natural Science Foundation of China (31960532, 32060604) and the Science and Technology Project of Yunnan Province, China (2018FD024, 202005AF150040).

## Conflict of Interest

The authors declare that the research was conducted in the absence of any commercial or financial relationships that could be construed as a potential conflict of interest.

## Publisher’s Note

All claims expressed in this article are solely those of the authors and do not necessarily represent those of their affiliated organizations, or those of the publisher, the editors and the reviewers. Any product that may be evaluated in this article, or claim that may be made by its manufacturer, is not guaranteed or endorsed by the publisher.

## Supplementary Material

Supplementary Figure 1 Symptoms on the tobacco K326 plants inoculated with TBTV-YK, TVDV-YK, TBTVsatRNA-YK, and TVDVaRNA-YK. The plants were photographed at 101 days postinoculation. The plant agro-infiltrated with pCB301 (an empty expression vector) was used as a control.

Supplementary Figure 2 Phenotypes of *Nicotiana benthamiana* plants inoculated with TVDV-YK, TBTV-YK, TBTVsatRNA-YK, and TVDVaRNA-YK at 16 dpi, 42 dpi, and 74 dpi, respectively.

Supplementary Figure 3 Symptoms on the tobacco plants inoculated with TBTV-YK. **(A)** The top view of tobacco K326 plants inoculated with TBTV-YK, arrows indicate necrotic spots in the systemic leaves. **(B)** The side view of tobacco K326 plants inoculated with TBTV-YK, rectangular dashed box indicate shorter internodes. The plants were photographed at 45 days postinoculation both in **(A,B)**. **(C)** Phenotypes of *Nicotiana benthamiana* plants inoculated with pCB301 and TBTV-YK at 74 dpi, respectively.
