# Supplementary material for: Synergism Among the Four Tobacco Bushy Top Disease Casual Agents in Symptom Induction and Aphid Transmission
Source: Front Microbiol. 2022 Apr 4;13:846857. doi: 10.3389/fmicb.2022.846857 (PMC9014100; doi:10.3389/fmicb.2022.846857)
Supplement: Supplementary file 1 [file Table_1.docx]

Supplement table 1. Virus sequences used for phylogenetic analysis

| Virus Name | Full Name | | | Accession Number | Lenghth（bp） | |
| --- | --- | --- | --- | --- | --- | --- |
| TBTV-YK2 | tobacco bushy top virus | | | OM062615 | 4152 | |
| GGGTTACGATATGGAGTTCATCAACAAGATAAAGCAATTGTTGGCAATGAATTTCAAGCCCTCTAAGGGCGTAATGTCTCGGGAGGAGCTCCGTGAAGCTTTCGATCCCACTTGGGAGCTCCTCATCACACAGGCTCGTGTCACCACTGAGGTGTCACGCCAGTGTGAGGATTGGTACACTCTAGCTGTACCCACCACCTACCGCTTGCCAGAGTTGGCGGTAGAAGAGGCTGTGCGTGAAAAGAACATAGCGCGCGAGGTGGCGGCCAAGTGCCCCCCTGAGGACCCGACTCCTGCTTCCCCGCAAGAGCCACAGCCTGTCCCCTTGGCGGAGAGCTCCGAGTGCTCGAGCCAGGATGCCGAGCGTGATGCGCTCGATGAGATATGGGGGCTCCCCACTCCCGTGCCACATCCTCTGCCCAAGTACTTCGAGCGGCGTTACCAGGCGCTGGCTCGGACCTGCCAGAAGGACTACTCCAACTGGCAGATTGTACCCTACACGGGTGGGCCACGCGTGCTAAGAGAGGAGGACGTGCTACCAATGGCCACGGGGGTCATACCCTTGCCACCCCCCCCCCCTAAGGCCTCTGTGATTGGAGCAGTCTTGGGGCTGATTACCCGCTTCACCAAGATGGCGGGGGAAGTGAAGGGCAGGCTGAGCGCACCGACGCGGGAGCCCTCGCCCACTTGCTACGGTCTAGAGCAGGTGGCTGGTGAGCCCATGGGCTACATGAATGCACACTCTGTAGCCATGGAGTTGCGGGCTAGGTACGGAGTTCAGCCCGCCACAGCTGCGAACTTGCAGCTCGGAAACCGGGTGGCCAGGGAAATCCTGGAAAAACAGTGCGGGGCCACCCGTGACATGGTGTTCATACTCGGGCACCTCGCCACCACCTTGTGGTTCACCCCCACTATGGTGGACTTGGCCCTTCAATGTGGGCCTAAGGATTTTTGCTAGGGGATGTGGTCGCTCGGAGGGGTGTAGAAACTAAAGTGAAGACGAAAATCCACCCCAAAATCCGAGTGCTTAGGGCGGCCCGTCCCCGGCCCGTAGAAAGAGTGTCGTACCAAATCGACGTGGTGCGGCCCTGTGCTGACTTTGGAGTCCACAACAACTCCCTCAACAATTTAGTGCGAGGGGTTAACGAGCGGGTGTTCTACACAGACCACAAGAGGAAAGAGCCCCGCAGACCTTCAGCTGGTAGTTTTGACAAGATCAACATCAGCGAAATAAAAGCGTTCAGAGTCCAGCCGTGGACTCTCGAGGAGGTCGTTGAGAGCTACACGGGTAGCCAGAGGGTGCGGTATGGACAAGCTGTTGAATCCTTAGCAGTAACGCCCCTCTCACGAAACGACGCCCGGGTCAAAACATTTGTAAAGGCGGAAAAGATAAACTTCACCGCCAAGCCTGACCCGGCCCCTCGTGTGATCCAGCCGCGGGATCCAAGGTTCAATGCCTGCTTTGCCAAATACACAAAGCCCTTGGAACCCCTACTATATAAGCAGCTGGGTAAGCTTTACCAGTTCCCGTGCATTGCTAAAGGCTTTAATGCCGTAGAGACCGGAGAGATAATAGCCAAGAAGTGGAAGTGCTTCAGTGACCCTGTCTGTGTGGGTTTAGATGCCTCCCGGTTTGACCAACATGTGTCATGCGATGCACTACGGTTCACCCATAGCGTATAtAAACGGTTCGTAAAGGGCAGGGAAGTGAACAAGTTGCTTTCCTGGATGTACAAAAACCACGCTCTGGGAAGTGCGAAGGACGGATTCGTCAAGTATGAGGTGGAAGGCTGTCGGATGAGTGGGGATATGGATACAGCCCTAGGGAATTGTGTCCTGATGGTCCTCATGACTAGGCAGCTATGCCAGAACCTCTCCATACCGCACGAACTGATGAACAACGGCGACGACTGCATAGTTATATTTGACAGGCAGTACCTGTCCACCTTTCAGGACGCAGTCGAGCCTTGGTTTAGGGAACTAGGGTTTACAATGAAGGTCGAGGAGCCAGTCTACCATCTCGAACGAGTAGACTTTTGCCAGACCCGTCCCGTGTATGACGGCAAGAAGTGGAGAATGGTCAGGCATATCTCAAGTATAGCTAAGGATTGCTGTTCAGTTATTGACTGGGAACAGTTACCGGCCTGGTGGAACGCCATTGGAGAATGTGGCATTGCTGTGGCTGGTGGCATACCCATACACAACAGCTTCTTGAGATGGCTCCTGAGATCAGGaGAGAGCAACCCTGATCTCCTGAAGCATGGCGCATGGAAAAATGAGGGCCTAGCGTGGTACCGGATGGGCATGGACCTATCCCATGAGAGACACGTTAGTGATGAAGCGCGCGCCAGCTTCCACACTGCCTTTGGAATCGAACCATCCATGCAGGTCGCATTAGAGCAGATCTATGACTCATTGCCTGCTCCCACCATTGGTGGGAAACGAGCCAGGGTGTGTAAACCTGGTGAAATGGTGTTGGTTGATTCACTCCCGCCGCGGCACTTTAATAATTACTTCCAGGATGTTGGAATaGGGGGGAGTAGCAGTGACTACGTTGTCCCGGGGGCCCATGAGTTCGAACCGGGCACGTTGTGGACACAATGCTAGTCGATCAAGTTAGCCAGGCATATGGTTGCAAGCGCTGGGGACTTAGTCCATAAAGCCACtGGTAAAGAGCCTCCAGTAGGCAGGCCCGCCTTGATGAGTATCAACGAGTGGACACCCGGGCAGCCGCGGACAATGCCCGACGGGTTTCAATGTCTACAATCATAAATGTCAACACTCATCCACAAGGCAGATACGAAAGAGGAGCTGCTCAACGCTCTATACGGGGAAGTGACGTTAAGGGAGCTCGAGGAATCAAACCTCGGCGTACTAACCCCCCACCGGGCCGAAAACAAGGTGGTAATGACCCCACTGCTGCCACCCAGAAGTCAAGGAAGAATCGCCAGCGTCCTGAAACGGTTCCGCCCCACAAAACACACGGGGGGACTTTTATTCATAGAGAAGATTGTGGTGGTGTTCACGCCCCACATACCGGACGATGCTGGCGGGGAGGTAGAAATCTGGGTCCACGACAACATGCTACCACACCTAAACAGCGTTGGGCCCCGCGTAAGATTTCCGATGAGCGGAGGGCCCAGGTTGATAGCATTTTATCCCCCCTACTCGATACCCTTAAGTTGTCAAGTGAGGGGGGCGCCCCGGAGCTACTTCATTGTATCGGAGTACTCCGGCGTGAACTTCGTCGCAGGCGCGAGCCCGTTCAGCCTGTACATAATGTGGGAGCCGAAGATAGAGTGCGTAGCGCACAACTATCTGATGAGGCCCCCCAAAGCGATACCGATTTGCAGACATCTGGTGAAGGACAGTCTGTCCTCCTTAACCCTAACTCAGGGGGCACTGAAAAGTGCAATGTCGAATCGGTATGCAACAACTGCGACCGGGCTCCCACCACTAACAAGTGGTGAGCAGGATATGGAGGTAGTGTCCCATCCTCCAGGCTAAATTGGACACAAAACGGAGCTAGATTTAGTGTTGCAATTGCTAGACCGTTAGTCACTACGCTAGACAGTGCGACAACTGAGGTCGTCTATGACCCACAGGGTGCCATCCTGTGCAATTTCTAACCCTTGGCAAGGGTGATCCTAGAGGACGCGGGCATTGAATTGCCTGGGGATGCTTCTAGTTACATACCGGAGTGGTTGATAGACCTCTGTGTAAGGAAACAGGTGTGAGACCGGATCCTGGGAAACAGGCTGGGTTGGTCCCGTAAGTTCGGGCGGTGCCCTTCTGGGGTAACCCCACCGCCGACGTATCACCTCACACGCTGTTGACCGACAAGCCGGGGTATTGGAACCGCACCACTCTGGAGGTTCCCTCTCCTTATGAGGTGGAGGTACAGCACGCCTTAAGTGTCTGCGACCCGGTGTTCACGTGCCGGTGGTCCCCCTAGTTAACGTGGTGCCACAGTCTGTGGTAGGGGTAGCTGGTCGGATTCCAGCATAAACCAAGTAGCAAGTGGACGTTGCTACCTCGAGCCTAGAGTCATGGGCCCGCATCGACGTTTGCGGTAACTCACAGCGGGCGATAATAGCTGTTGAGAATGTCGGTTCCTGACTTAGCGGGAGATGAGCACTCTCTCTCGCGCCC | | | | | | |
| TVDV-YK2 | tobacco vein distorting virus | | | OM062616 | 5918 | |
| ACAAAATATAAGAAGGGAGAGACCCTTGCTCGAGTTTTCTTCGAAGCTTATGAACTTTGAATTGATCAACGGAAGCCATTTGAAAGTTTCCACAACTCGCAAGCTATCTTACAAAGAGAGACTTTTGAACTTAGCTATTTTCCTGAGTCAATTTGTCAATAAATTCGAGGAAAATGCAACTACGAACACTTTTCTTCGTTCTCTTTGCGCTCTTCTCCCTCTCCTTCTCAGCAGCAGATGTCCCTTCTTTTCAGGGAATGCCCCGCACCGGAACAGCAAGCGGGAACGAAAGCGGATTTCACGGCTCGCTATTTATAGTGGAGCCAATATCACTCCCTGCTGTAGAGAAGAGATCTCTTCCCCCACCTGGCTCCAAACCGATAATAAACTCGGAGCCCCACGAGCCCACCTTAAGAGATGCGCTAGTTCTGCTTTGGCAAGTTACCTCCAGAGATTCCAGACGCCTCTATTCCAAGGCGCAGAACAGTTTAGTAAGTTCTTGCGCGTCTGGACTCACCATTGTGAAAACGTGGTCACAAGGAGCTTTCCGAAGCTTCCTCTGGGCCGTGGTCTCAATTTGGAGCTTGAGCATTTGGGTCATTGTCTCCTGGATCTTCTATCTGGTGACGACATTTACCATGCCCGTCGTATGTCTCGTATTGCTCTATGCCTTCACAACATTTATGGTGAAGGCGTTGCAGTGGATGTTTACCGGCTGGCCCACTTGCCTCGCTCTGCTTATCCTAAGAGTGGGGAAGACTATCTTCACGGTTCCCAGATTCAAAAGGAGCTACAGTGAAGAGAAACAGGTTAAGGGATTTGTCTCCTTGAAAATCCCCCAAAGCCCCCCCCGTGGCTGTGTGCTACTCGTGCAACATGAAGATGGATCTCATGCCGGGTATGCCAGTTGCGTCAAGTTGTTCGACGGCACTTTAGCCCTCATGACTTGCCACCACGTGGGCACAGGGGTTCCCAAAGGAAAGGTGGCTTCCTCAAAGACCACTAACAAAATACCACTCAGCCTATTCACACCTCTGATCTCTTCCGAAAAAGGAGATTTCATGTTAATGAGCGGACCACCAAACTGGGAGAGTCTGCTCGGCTGTAAAGGAGCCACCTTTGTTCCCGCATCACAACTTGCTAAATCCAAGATGCGGTTCTTTTTCATTGAGAAGAACGAATGGATGGCTGATCACGGAGAGATTGTAGGTCCCCGTGATCACTGGTTTGCTACCACACTCTGTAACTCAGAGCCCGGACACTCCGGCACCCCTATCTTCAATGGTAAATCCATAGTGGGTGTGCATGCAGGAGGAGAAAATGAGCAGAACTTCAATGTAATGTCAACGATCCCTCCCATCCCCGGTTTGACGACCCCGCAGTACGTCTTTGAAACTACTGCTCCTCAAGGTCGGGTCTTTACTGATGAGGACCTGGGTGAGATGCTGCAAAGCGTTCACTCATCCGTACCCCAGCTGCATAAATTTTTGAGCAAGACTGGGAAGAACTGGGCGGATTATTCCGACGAAGAAGATTTTAGTGTGGAAATCAAAAACGAACCAGTCCAGGAGCCCGCGGCAGAAACAACGCCGCCTCAACTTGATACTATAGAGGCTCCGCCGGTGTCTGAATCCCGGCAGTGGTACCCTCGTCTTCCTGAGACGGGAAACGAGAAGGGCAGAGCTGTCTGCCAAAACAACAGCACCCCAGCCTCAGCACAAAACAAGGGCAAGGAAAAAGAGGTGGTGGCCGAAAAGGCCCCCGCAAACTCCACCCAGCCGAAGCTTCCAGAATCAGCCGACGGCGGCAAAGAATGTTTAGAGAAGCTTTTGGAGAAAATGGTGGAAAGGATCGACCTTTCTGCTATAGAAAAGAAAGTGGTGGAAGTATTAGCCCAGAAAGCCATGAAGAAGCCTCGAGGATCCCAGCGGCGCAGACGACCGCAGAAAACTTCCGACGATATTTCGAAGGAAAGTACAAATGGGAGGTACCAGCCTCCCCACAAGAGATCCCCGGCTTTGAAGAATGCGGAAGCCTCCCCCAGTACTACCACCCAAAGCAAATCAAAAGTAGTCAATGGGGCACCCAACTCATCCAAGAATACCCGGAGCTGGGTGAAAAAGTCTCCGGCTTCGGCTGGCCGTCAGTAGGCCCCCAAGCTGAGGTGACCTCTCTCACTTTACAAGCAGAGAGGTGGCTGCAACGCGCGCAGTCAGCTAAAATCCCGTCATCTGAGGACCGGGAGCGCGTTATTAACAAAACAGTGGAGGCTTACTCAAATGTTAAAACTTATGGCCCTACTGCTACTAGAGGAAATAAGCTCGAGTGGCGGCAATTCATTGAAGATTTTAAGTCAGCCGTCTTTTCTCTGGAGCTTGATGCAGGTATAGGCGTACCATATATCGCCTATGGAAGGCCCACTCATAAAGGTTGGGTGGAAGACCCGAAACTCCTGCCAGTGCTTGCCCGACTCACTTTCAACCGACTACAGAAGATGTTGGAAGTTGAGTCTTCTGAAATGAGTGCTGAAGAGCTTGTTCAAGCTGGTCTCTGTGATCCTATAAGGACATTTGTAAAGAGAGAACCGCACAAGCAATCTAAACTCGATGAAGGCCGCTACCGCCTCATCATGAGTGTTTCCCTAGTAGATCAACTGGTAGCCCGGGTTCTGTTCCAAAATCAGAACAAGCGAGAAATCGCTCTTTGGAGGGCAAACCCCTCAAAACCCGGTTTTGGCTTGTCTACGGATGAGCAAGTGCTGGAGTTCGTACAAGCTCTGGCCGCGCAAGTGGAAGTCCCACCTGAGGAAGTGATTACCTCCTGGGAGAAGTACCTTGTGCCGACTGACTGCTCTGGTTTCGACTGGAGCGTTGCGGAATGGATGCTACACGACGATATGGTCGTCCGCAACAAACTCACATTGGACCTGAATCCGACAACGGAAAAGCTGCGCTTTGCGTGGCTAAAATGCATTAGTAACAGTGTCCTTTGTTTGAGCGATGGCACCCTGCTAGCCCAAAGAGTCCCCGGTGTTCAGAAATCTGGGAGTTACAACACAAGTAGCTCTAACTCCAGAATCCGGGTTATGGCCGCTTATCATTGCGGAGCCGACTGGGCCATGGCCATGGGAGATGATGCCCTCGAGTCAGTCAACACCAACCTAGAGGTGTATAAAAGTCTAGGTTTCAAAGTCGAGGTTTCAGGACAACTGGAATTCTGCTCTCACATTTTTAGAGCGCCTGACCTCGCCCTCCCAGTGAATGAGCGTAAAATGCTGTACAAGCTCATCTTCGGTTACAATCCTGGGAGCGGGAGTCTGGAGGTGATCTCCAACTATATTGCTGCCTGTGCATCTGTGTTAAACGAGTTGCGGCATGACCCAGACTCAGTTGCTCTGCTCACCCAGTGGCTAGTCCATCCAGTGCTGCCACAAAACGATTAAAGGAGAAAGCAAGAAAACTAGCCAAGCATACATCAGTTGCAAGCGTTGGAAGTTCAAGTCTGATTACCAAAGCCCGACACCATAGATTTTAAATTTTTAGCAGGATTTGCGTCAGGATTTCTATCCGCAATCCCAATTTCAGTTGCGGGCATTTATTTAGTCTACCTTAAAATCTCAGCCCACGTTAGAGCTATTGTTAATGAATACGGGAGGAGTCAGGAGTAATAATGGAAATGGTGGATCACGAGTCTCTCGCCCTCGCAGACGCGCACGATCGGTTCGGCCGGTCGTTGtGGTCGCACCCCCTCGGGGAGCACGGCGAAGAACTCGAAGAAGACGAAATGGAGGCAGGAACAGAAGAAGCCGTAATGGAGTTGGAGGAAGGTCAAGCAACAGCGAGACTTTCATCTTCAACAAGGACTCAATCAAGGATAGTTCCTCAGGCTCAATCACTTTCGGGCCGTCTCTATCAGAGAGCGTCGCGCTTTCAGGTGGAGTTCTCAAAGCCTACCATGAATATAAGATCACAATGGTCAACATACGCTTCATCAGTGAATCCTCTTCCACAGCGGAGGGCTCCATCGCTTACGAGCTGGACCCCCACTGCAAGCTTTCTAGTCTCCAATCAACCCTCCGTAAATTCCCCGTCACCAAAGGCGGGCAAGCAACGTTCAGGGCTGCGCAAATTAATGGGGTAGAGTGGCATGATACAACTGAAGATCAATTTAGGCTGCTCTATAAAGGCAACGGAACAAAAGGTGTTGCCGCCGGGTTCTTTCAAATCCGGTACACCGTGCAACTGCACAACCCCAAATAGGTAGACGCCGAGCCAGGACCTAGCCCAGGACCTCAACCTCAACCCACACCCTCACCTTCTCCTCAAAAGCATGAGCGCTTCATTGCATACGTCGGCATTCCAATGTTGACAATTCAAGCGCGGGAGAACGACGACCAGATCTTATTAAGATCCATGGGTTTGCAACGGATGAAGTATATAGAGGACGAGAATCAAAACTATACGAATATCGATTCGCAGTTtTATTCTCAGTCTAATGTCAATGCCGTCCCCATGTATTATTTCAACGTGCCAAAAGGTACATGGTCAGTCGATATCAGCTGTGAAGGTTATCAGCCAACGAGCAGCACCACGGATCCAAACCGTGGGAGGAGTGATGGGTTAATAGCTTATTCCAATAGTGACTCAGATTACTGGAACGTAGGAGAAGCGGATGGTGTTAAAATTTCAAATTTAAGGAATGATAACACGTACCGCCAAGGACATCCAGATCTTGAGATCAATTCCTGTCATTTCAGAGACGGGCAACTGTTAGAACGTGATGCAACTATAAGTTTCCACGTCGAAGCCCCAGATGATGGGCGTTTCTTTCTGATAGGACCCGCGATTCAGAAAACCGCCAAGTACAATTATACAATATCTTATGGCGAGTGGACAGACAGAGATATGGAATTAGGGCTAATCACAGTAGTGCTAGATGAGCACCTTGAGGGTTCTGGCTCGGGCAATTTGGCGAGGAGAGAAAGGAGATCATTGCATCAACATTGCAAATCTCTCTACGCCCCGGAAGGACCACCGGGAAACAAACCCTGGGATGACAACCAGATCATTGTTGGAGAAAGACAACTCCGTAAAACTCTGTCTATGGACATGTCAGAAACTGGTTCTGACGTGAGAGAAGGAAAACTCCAATCAAACCCTAGTGGTGATGCCAGGGATGAGTTGAATTACACGTTGAGTCTTAGTGACTCCGAAGATGAGCTCATTGACGAGCCAAACTTCGTTCCTGGAATGCGGATGAATTTAGAGGCAGTACCTTTAGCTAATCCCGCTGACAAAGACTACATACTTCGCGACAGAGGCATAGCAACGACTTTGGTTGAACCAGCCTATATGCAAGGTGTAGCTCCTCAGAACAAAATTGAGGAGGATCCCTGGAAACATGTCCGGGATTTCAAATCTGCTCACACTAGTAAAGGCCCGGCTTCTGTAGCTTCTGGGTCTACAATGAGAGGTAATTTAAGAGGAGGTACTTTAAGAGGAAGATCTATGCCTCCACCTCTCCCCCCTCCAGCTGAGAAAGTTGGGGACTCCCTGCCTTCGGGCAAACTTCAATGGGATCCCCCAGGCTCGCCCCTTACTCCTTTTAAGGATAAGGAACTGGCTAAACCGCCTTCTGATGCTCGTAGCTCGATTTCTGGTCGTCTGACTGGAGGTCTTCTGAAAAGCCGAGTAGATAAAGATTTGGCGAGTCTGACCACAGCTCAAAGACAGCAGTACGAAATAACTAGACAAACTCTTGGTAAGGTTGCTGCTAAAGAGTTCTTACAAAGATGTAAGGAGGGTGGTTCATAGTCTAAGCTCTACGACTATAAAATAACGAGCACACTGAACTCCAAGCCTGTTAGAGTATAACCCAGGCAGTCTGAACTCTAAGACTTTAAAACTGTGAGTAATCCT | | | | | | |
| TVDVaRNA-YK2 | tobacco vein distorting virus associated RNA | | | OM062617 | 2971 | |
| GGGGATTCATGGAGACAGCTAGAGGTTTACTAGACGCCCTAGGCGATCCATGCACCAACGTGGTTCGGGCTTTCAAGGCACGACCTGTCATATACAGCTTTGACCAGGTCGTACATGCCTTCTCAGCTGAAAACCCCTTCTCAGGTTGTAGTGAGAGGGTGGTGTTAGCTGTACCCCATGTCATTATGGGGAATAGTCCATTAGAGCGGCTATTGGAGCTGCTCGCCACATCTAGGATAGCGGTGGCTCAGTTTAAACGGTCTATAGTAAACTGGGTTCACTGTAATAGTATATATTTAGGATTGTCGGCTGCAACTAGTGCAGCCGCCCTAGTAATAGTAGAGAGATGGAGGCTTTCCCAAGCCCCCCCCATGCAAGAGGAGCCAGTGCGATTGGAGGATTGCCTGGAGATAGATGATCCTGACCTCCCCCTCCTGGAACAAACTGTGCCAGATGAGCCAACCATTGACAGAGCTAGAGCTCAGCTGGCTATCCGAAAGAGGGTTACCCGTAGAGTGAGGCCCAACTCAACAGCTAGATTCATAAGGGTCCTCAGAGCCGAGGTGAAAGCGACTGTCGGAACACCAACATACACAGCTGCGAACGTGGCTGTAATCCGGCACATTGTCGAGAAATTCGCGAAGGAGTACAACATTAGAACATCCTCGTACTCCCACCTCATGGGCGATGTGATAAGCGCAGTCATGACTCCTTACAAATCGGAGTACAGGCAAGCTGCGTACTGCGGGAGTCTCACGAGTCGCCTACGGCGGTGGCTCGTTGAGCTTAAATAGGGGGGCCTACAGCTGGTGGGTGGGTACGAGCATGATATGCCAGATCTTACAAACGTACCCGGCCTGGACCCACGACAACTGAGGCCGTCTATAGTAGAGTATAACAAGGAATTCCGTCGTATAGTGGCACCTAGCTACAACTACGGAGTGACTTATTTTAATAATAGTAGAGCTAACCTACTGAGGGGTCTTGTCAACAGGGTGTTGACATATAAGGGTGGACCTATCCTCGAGCCTAGTCCGGGCTCCTGGAAGTCACTTTCCCCGCTCGCAAGGAAGATAGGTATTAGGTGCAGAGCCTCCCCGATGAGCCCGGATGAATTTCTGGCGTGCTATGTGGGACGTAAACGCACTATCTATACCAAGGCCCTTCGCAGCTTGGAGGACACCCCCCTCAACAATAAAGATTTTGTGGTTCGGGCCTTCTTAAAGAAGGAGAAAGATAAACTGCTGAGTAGCAGCTCCGATCCCAGAATAATTCAGCCTCGACATCCGAGGTTTTTAGTTAGTTTTGGTAGATACATCAGACCTCTGGAACAGAAATGCTATAAGGCATTCTCATCGTTGTTCCAGCGATTTGCCGTGTCTCGGACCCCTGTTTGCTTTAAAGGTCTCAATTACTTGCGCAGGGGCGAGATGCTCTTAAAGAAGTGGGAAAGCTTCGTTAACCCCGTGGCGATTTGCTTAGACGCATCTCGTTTTGACCTCCACGTGTCGGTTGAAGCACTCCAGTACACCCACATATTGTATTACACGGCATTCGGTAGGGATCCCTTGCTCATGCAACTATTGGAGAACCGCTTGGAAACCCGCGGAGTAGCTATTTGCAAGGAAGGATCCTACAGGTACAGGAAGAAAGGTGGGCGTTGCTCAGGGGATAATGACACATCTTTAGGCAACGTGATTATAATGCTATCTATAACCTACCATCTTCTTGAGGGACTGCCTTTTCCACACATTGAAGTCGCTAACGACGGAGATGATCAAGTGCTCATTGTGGAAAAAGAGAATGAGTTAGCCCTGCTGGAGGCAGTTGAGCCCTGCTTCGCGAAATTTGGGTTCCGGCTAACCGTCGAACAGCCAGTCTATGAATTTGAGCGCATAGACTTCTGCCAGACTCGGCCTGTTCGATTGAGTCCCGATGTGACAACTATGGTCCGACATCCACGCCTCGCCATGACGAAAGATTTAACCTCCTTCATCCCAATAGAACGAGGGTTGATGAAGTACCACATGCTAAGCGCAATAGGCGCATGTGGTAAGGCTTGTTATAATGATATACCCGTGCTCGGAGCAATGTACAGAAAGCTTGAGAGCGTGGGACGAGCAGCCGGAGGCAGAGAAGAAAAATGGTGGGCGTCAACGAATGCAAACCCCGACTTTCAAAGATTATCAAGCAAAGCAGCGACCAACGTAGGACTTACTGTTGAAGCTCGCGTATCGTTTTGGAAGGCATTCGACATTCTCCCCGATCACCAAACCGCTCTAGAAAGAGAATGGGAGAATTGGAAACCGGACTTTGAGTTGGCTACCGTTCCTGTTTTTGTAGGAACCTATGCTGTAGTAGATGGTGATGTTTAAAATTAGACCAGTCGCCCCCGTCCTCCGCTGGATGGGGGGGAAGACCCGTGGCCGCGGTGTTATGCTAGGAACTCCCACGTAAGTGGATCGGCCCAAACCATCGAAGTAGCATAGTTATTATAATGTATATTTAGTCTAGTTTAGGATCGAGTCTTAAGGCAGCCCCTTGCCAGGTTGCGACTAGGGGGCCATCAGGCCTGGCACACCCGTTGGTTAGCTCGTAACTACTCTCGTTGGATCGAGAGGACAATCCGAGTAGAGGCAGAGAAGGGCCTCCGGTTTGACCATACCGTAATAGCCGGACGCATAGCTATTCGGGCTGGGGACGTCATACCCAGTGCGATAGGTCCCGCAGTTGGACTAGGACACTTGTCCCGGCCGTTTCTCTAAGTGGCGCGCTTCAATCAGCAAACTCTTCCGCTAGAGTATTGCTTAGTACTAGGCTCTTAGCCAACCTCTTTCAATGTAAGAGGACAGAAGCGTTTTGGTCGGAGGAGAGACCGTCGCACCTGAACTCCATCACTGGGAAACTCCAGTGAGGGCGATAGGAGATTCAGGAGGTTTGGAAACACTTTGTTTACCAGGTACACCGCCCC | | | | | | |
| TBTVsatRNA-YK2 | | tobacco bushy top virus satellite RNA | OM062618 | | | 824 |
| GGGTATCGATACAAGGAGCAGCATGCTCATTCCTCTTAGTTGGAGACTGTGCGGAGGAGTATGCATCTTTTTGGACGGAGAGATGTTGGCCGTGGGCAGCAACCCGTGGATAGCTGATAAGATCGTCCAGTGGATGGGGACCTAAGGACCCCCATTGGCTCTCTCTCTTCTGTCAGAGCGGCTTGGTGAGCCGCCAGAGTGAACCGCTCGAGCTGAATTAAAACCCCTAAGCGGTTCGCTCTGGAGCCTGTAGAGTCCACGTCCATCGCCCTTAGTTGGAGACTGTGGGTGCTTGGCGTAGCCCTAGTAGTGGCACGCCTTGAAGCTCCGATACCTATCCTATTAGCCCTGGCCGTTGATACTTGTGGATCCAAGAGGAACCACTCGAACGAATCGGATCACCAGGGAGTACCCTTAGGGATGGACCAGCAACCAACTGGGCATCAACGGGATCGGCTAGACGTAGGACAGCAGTAGGCGGGAGACACTAACGCCGCGCCTCCTACGGCTCTGCGTAAGCAGACAAGTGAGACTCACCTCAGTCACCGAGTACCTGGATTAAGGGGAAAGGAGGGAGTCGATTACCGCGTCCCCGGCTGGCATAACGCGAGCCCCGCGGTAGGAGTAATAGTGCAGTGGTTAGGAAAGCTGTGCGGCGCCGACGCGACGGAGCCAAATACGGAGGACTGTGGCTAGGCTGCCTTTGGCCGATGCCGTCATGCACTGGGGTAGTACCCCAGCCCAGAACACCCTGGGGAGTCTCCCTCCACTCGCGGTGCCCTGAAACGGGCACTACTACCTAATAAGTAGATCCCATCTAGCCC | | | | | | |

Supplement table 2. Primers used in this study

| Objective | name | sequence |
| --- | --- | --- |
| One step multiple RT-PCR ^a^ | TBTV-YK dF | 5′-TACCACACCTAAACAGCGTTG-3′ |
|  | TBTV-YK dR | 5′-CTCATCTCCCGCTAAGTCAG-3′ |
|  | TVDV-YK dF | 5′-GCAACAGCGAGACTTTCATCT-3′ |
|  | TVDV-YK dR | 5′-CRTTGCCTTTATAGAGCAGCC-3′ |
|  | TBTVsatRNA-YK dF | 5′-TGTTGGCCGTGGGCAGCAA-3′ |
|  | TBTVsatRNA-YK dR | 5′-TTCGTATTTGGCTCCGTCGC-3′ |
|  | TVDVaRNA-YK dF | 5′-ACTCCCACCTCATGGGCGATG-3′ |
|  | TVDVaRNA-YK dR | 5′-TGTACTGGAGTGCTTCAACCG-3′ |
| qRT-PCR detection ^b^ | TBTV-YK qdF | 5′-TCGAGGAATCAAACCTCGGC-3′ |
|  | TBTV-YK qdR | 5′-GCGTGAACACCACCACAATC-3′ |
|  | TVDV-YK qdF | 5′-CCGTAAATTCCCCGTCACCA-3′ |
|  | TVDV-YK qdR | 5′-ACACCTTTTGTTCCGTTGCC-3′ |
|  | Actin dF | 5′-CTGAGGTCCTTTTCCAACCA-3′ |
|  | Actin dR | 5′-TACCCGGGAACATGGTAGAG-3′ |
| RT-PCR detection of virions ^c^ | TVDV-YK-CP-dF | 5′-GAGTCTGGAGGTGATCTCCA-3′ |
|  | TVDV-YK-CP-dR | 5′-CAGCTCGTAAGCGATGGAG-3′ |
| One step RT-PCR and RT-PCR detection of virus by the acquired and transmitted aphid ^d^ | TVDV-YK dF(852-871) | 5′-CGTGGTTGTGTGCTACTCGT-3′ |
|  | TVDV-YK dR(1581-1600) | 5′-AGTTGAGGCGGCGTTGTTTC-3′ |
|  | TBTVsatRNA-YK dF1 | 5′-GCAACCCGTGGATAGCTGAT-3′ |
|  | TBTVsatRNA-YK dR1 | 5′-TAGTATCTTCCGCCGGTTGC-3′ |
| The full-length cDNA colony PCR ^e^ | TBTV-YK IF | 5′-GTTCATTTCATTTGGAGAGGRGGTTACGATATGGAGTTCATC-3′ |
|  | TBTV-YK IR | 5′-GTGGAGATGCCATGCCGACCCGGGCGCGAGAGAAAGTGC-3′ |
|  | TVDV-YK IF | 5′-GTTCATTTCATTTGGAGAGGACAAAATATAAGAAGGGAGAGACC-3′ |
|  | TVDV-YK IR | 5′-GTGGAGATGCCATGCCGACCCAGGATTACTCACAGTTTTAAAGTCTT-3′ |
|  | TBTVsatRNA-YK IF | 5′-GTTCATTTCATTTGGAGAGGGGGTATCGATACAAGGAGCAG-3′ |
|  | TBTVsatRNA-YK IR | 5′-GTGGAGATGCCATGCCGACCCGGGCTAGATGGGATCTACTTAT-3′ |
|  | TVDVaRNA-YK IF | 5′-GTTCATTTCATTTGGAGAGGGGGGATTCATGGAGACAGCT-3′ |
|  | TVDVaRNA-YK IR | 5′-GTGGAGATGCCATGCCGACCCGGGGCGGTGTACCTGGT-3′ |

1. The One step multiple RT-PCR detection primers were used to detect the virus in the inoculated plants, TVDV-YK and TBTV-YK primer of which also were used to detect the aphid transmission of virion, and acquisition or transmission efficiency of virus by the aphid, respectively.
2. The qRT-PCR detection primers were used to identify the viral RNA level and the Actin were used as control in *N. benthamiana*.
3. The RT-PCR detection primers were used to detect the RNA of purified virions.
4. The One step RT-PCR and RT-PCR detection were used to detect acquisition and transmission efficiency of virus by the aphid.
5. The full-length cDNA colony PCR were used to generate infectious clones (pCB301-TBTV-YK/TVDV-YK/TBTVsatRNA-YK/TVDVaRNA-YK).

Supplement table 3. Aphid transmission of purified virions of tobacco vein distorting virus after in vitro acquisition via membrane feeding

| Virion concentration(mg/mL) ^a^ | Transmission by *Myzus persicae* ^b^ |
| --- | --- |
| 20 | 1/3 ^+^ |

1. Virions were purified by the standard procedure combined with an additional ultracentrifugation through a 10–40% sucrose gradient after *N. benthamiana* inoculated with TVDV-YK 14 days, and virion concentration was estimated from absorbance values at 260 nm.
2. On *N. benthamiana*, transmissions were performed using *Myzus persicae* feeding on purified virions of the indicated viruses.

+. Number of plants infected virus after *Myzus persicae* inoculation /total number of plants tested after *Myzus persicae* feeding on purified virions.
