# Supplementary figures and images for: Synergism Among the Four Tobacco Bushy Top Disease Casual Agents in Symptom Induction and Aphid Transmission
Source: Front Microbiol. 2022 Apr 4;13:846857. doi: 10.3389/fmicb.2022.846857 (PMC9014100; doi:10.3389/fmicb.2022.846857)

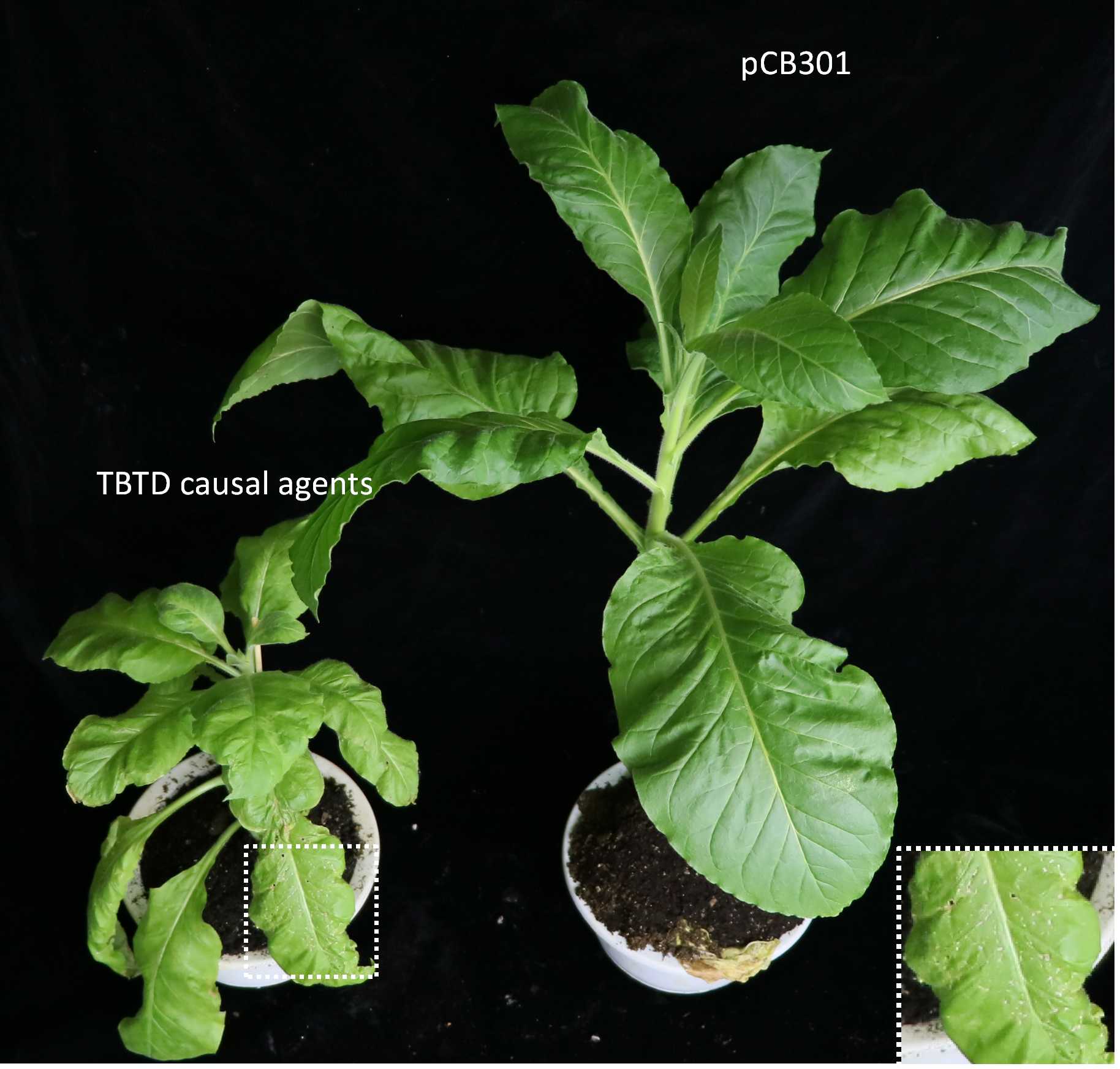

Supplement: Supplementary Figure 1 — Symptoms on the tobacco K326 plants inoculated with TBTV-YK, TVDV-YK, TBTVsatRNA-YK, and TVDVaRNA-YK. The plants were photographed at 101 days postinoculation. The plant agro-infiltrated with pCB301 (an empty expression vector) was used as a control. [file Image_1.jpg]

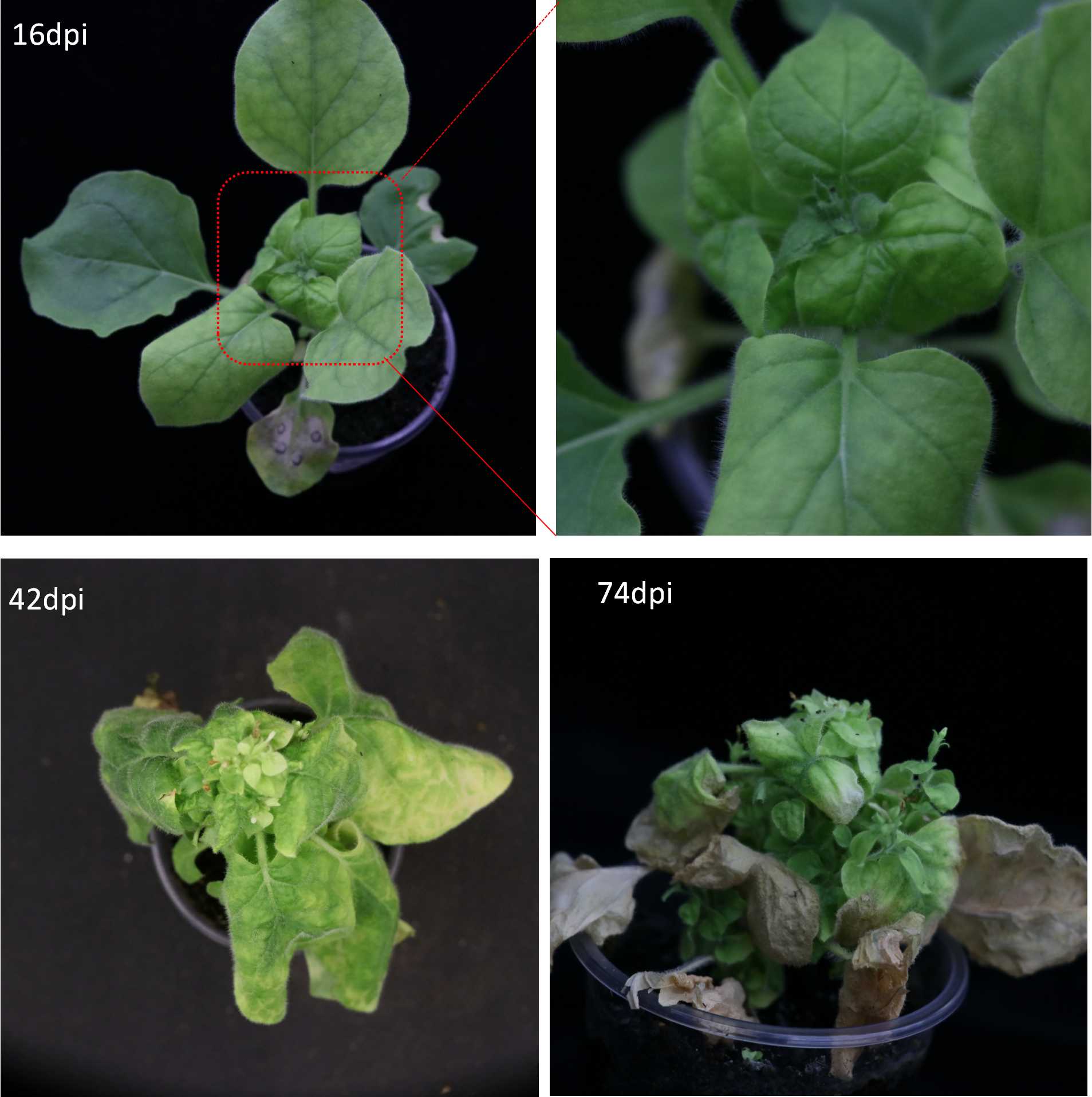

Supplement: Supplementary Figure 2 — Phenotypes of Nicotiana benthamiana plants inoculated with TVDV-YK, TBTV-YK, TBTVsatRNA-YK, and TVDVaRNA-YK at 16 dpi, 42 dpi, and 74 dpi, respectively. [file Image_2.jpg]

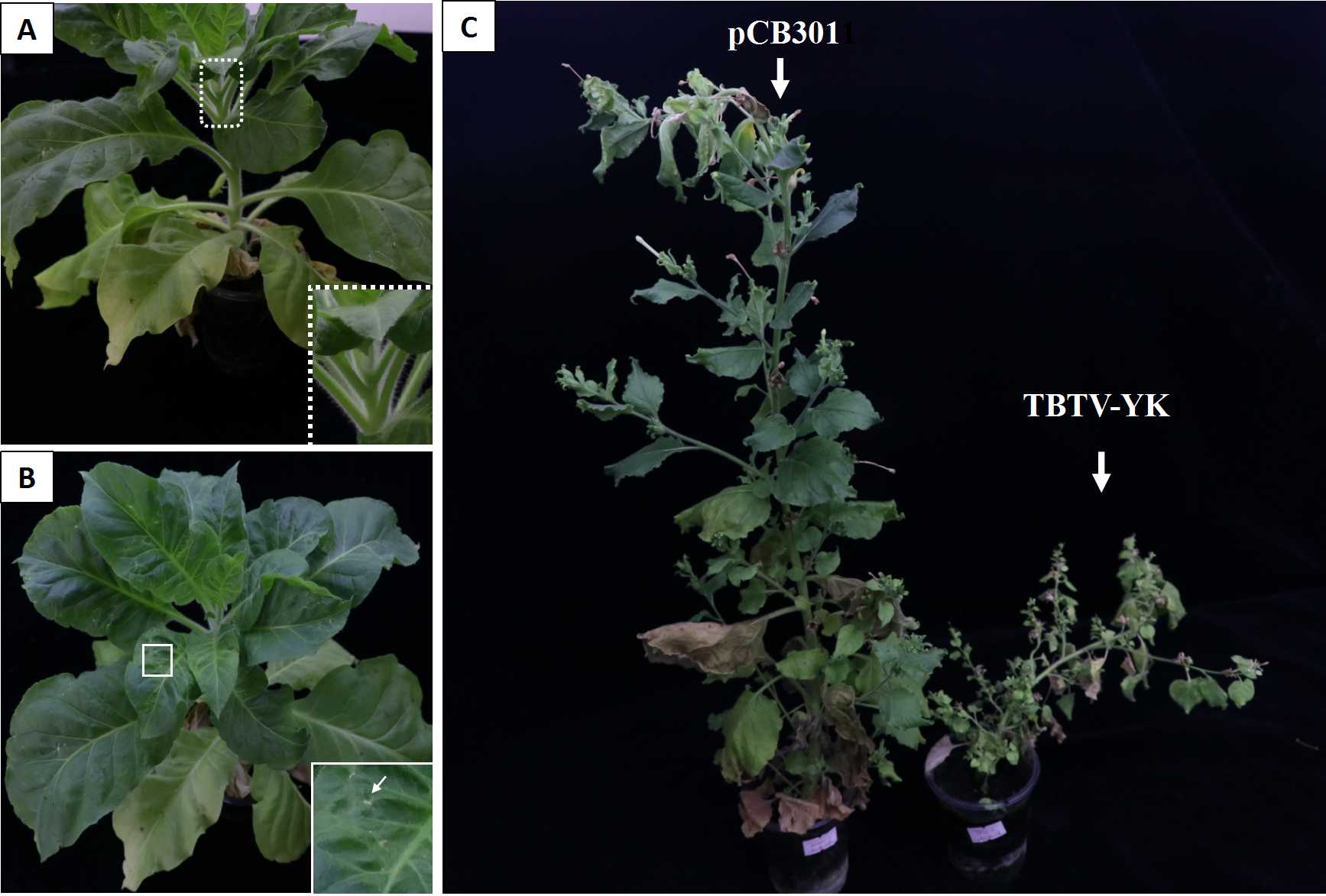

Supplement: Supplementary Figure 3 — Symptoms on the tobacco plants inoculated with TBTV-YK. (A) The top view of tobacco K326 plants inoculated with TBTV-YK, arrows indicate necrotic spots in the systemic leaves. (B) The side view of tobacco K326 plants inoculated with TBTV-YK, rectangular dashed box indicate shorter internodes. The plants were photographed at 45 days postinoculation both in (A,B). (C) Phenotypes of Nicotiana benthamiana plants inoculated with pCB301 and TBTV-YK at 74 dpi, respectively. [file Image_3.jpg]
